# Three-Dimensional Modelling of Ovarian Cancer: From Cell Lines to Organoids for Discovery and Personalized Medicine

**DOI:** 10.3389/fbioe.2022.836984

**Published:** 2022-02-10

**Authors:** Christine Yee, Kristie-Ann Dickson, Mohammed N. Muntasir, Yue Ma, Deborah J. Marsh

**Affiliations:** ^1^ Translational Oncology Group, School of Life Sciences, Faculty of Science, University of Technology Sydney, Ultimo, NSW, Australia; ^2^ Northern Clinical School, Faculty of Medicine and Health, University of Sydney, Camperdown, NSW, Australia

**Keywords:** ovarian cancer, 3D cell culture, 3D bio-printing, organoids, tumoroid, drug screening, personalized medicine

## Abstract

Ovarian cancer has the highest mortality of all of the gynecological malignancies. There are several distinct histotypes of this malignancy characterized by specific molecular events and clinical behavior. These histotypes have differing responses to platinum-based drugs that have been the mainstay of therapy for ovarian cancer for decades. For histotypes that initially respond to a chemotherapeutic regime of carboplatin and paclitaxel such as high-grade serous ovarian cancer, the development of chemoresistance is common and underpins incurable disease. Recent discoveries have led to the clinical use of PARP (poly ADP ribose polymerase) inhibitors for ovarian cancers defective in homologous recombination repair, as well as the anti-angiogenic bevacizumab. While predictive molecular testing involving identification of a genomic scar and/or the presence of germline or somatic *BRCA1* or *BRCA2* mutation are in clinical use to inform the likely success of a PARP inhibitor, no similar tests are available to identify women likely to respond to bevacizumab. Functional tests to predict patient response to any drug are, in fact, essentially absent from clinical care. New drugs are needed to treat ovarian cancer. In this review, we discuss applications to address the currently unmet need of developing physiologically relevant *in vitro* and *ex vivo* models of ovarian cancer for fundamental discovery science, and personalized medicine approaches. Traditional two-dimensional (2D) *in vitro* cell culture of ovarian cancer lacks critical cell-to-cell interactions afforded by culture in three-dimensions. Additionally, modelling interactions with the tumor microenvironment, including the surface of organs in the peritoneal cavity that support metastatic growth of ovarian cancer, will improve the power of these models. Being able to reliably grow primary tumoroid cultures of ovarian cancer will improve the ability to recapitulate tumor heterogeneity. Three-dimensional (3D) modelling systems, from cell lines to organoid or tumoroid cultures, represent enhanced starting points from which improved translational outcomes for women with ovarian cancer will emerge.

## 1 Introduction

Globally, ovarian cancer is the eighth most frequently diagnosed malignancy and cause of cancer-related death in women (Sung et al., 2021). The classification of ovarian cancer includes distinct histological subtypes with varied sites of origin underpinned by defining molecular events affecting tumor suppressors and oncogenes. These events drive specific patterns of clinical behavior characteristic of histotypes, including response to chemotherapeutic agents and molecular target drugs. Malignant histological subtypes arising from epithelial cells include high-grade serous ovarian cancer (HGSOC), ovarian clear cell carcinoma (OCCC), endometrioid ovarian cancer (EnOC), mucinous ovarian cancer (MOC), low-grade serous ovarian cancer (LGSOC) and malignant Brenner cell tumors (Shih Ie and Kurman, 2004; Kurman and Shih Ie, 2010). Ovarian carcinosarcomas (OCS), also known as malignant mixed mullerian tumors (MMMT), have epithelial and mesenchymal components (Harris et al., 2003). Ovarian sex-cord stromal tumors (SCST), the most common of which are granulosa cell tumors (GCT) along with the rarer Sertoli–Leydig cell tumors, are of stromal cell origin (Fuller et al., 2017). An extremely rare subtype of ovarian cancer primarily affecting women under 40 years of age is small cell carcinoma of the ovary, hypercalcemic type (SCCOHT), an ovarian rhabdoid tumor (Auguste et al., 2020).

**FIGURE 1:**
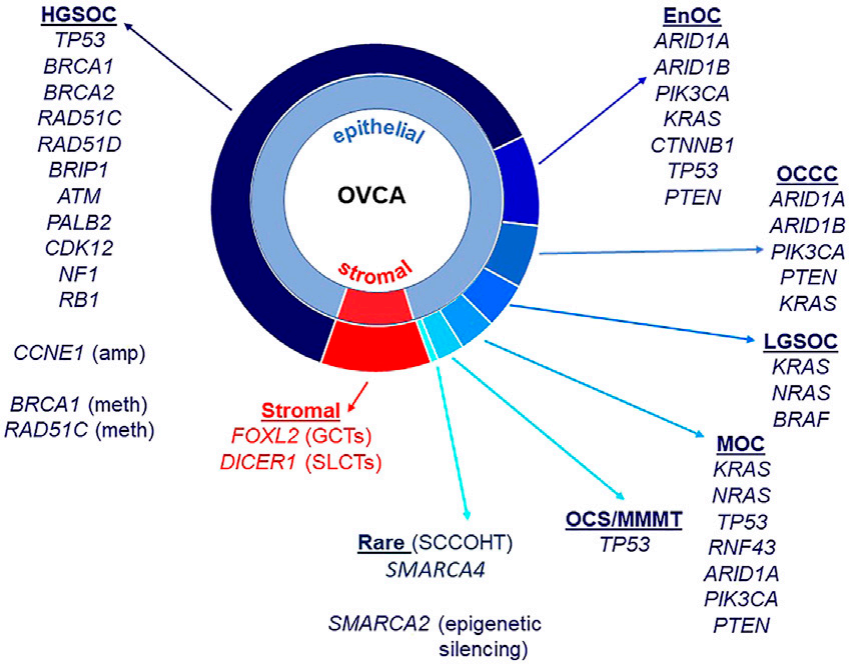
Ovarian cancer histotypes and gene mutations. Epithelial ovarian cancers constitute approximately 90% of all malignant ovarian tumors and are made up of different histotypes: high-grade serous ovarian cancer (HGSOC), endometrioid ovarian cancer (EnOC), ovarian clear cell carcinoma (OCCC), low-grade serous ovarian cancer (LGSOC) and mucinous ovarian cancer (MOC). Ovarian carcinosarcomas (OCS)/malignant mixed mullerian tumors (MMMT) have epithelial and mesenchymal components. Stromal cell tumors include granulosa cell tumors (GCT, adult and juvenile) as well as Sertoli-Leydig cell tumors (SLCTs). Small cell carcinoma of the ovary, hypercalcemic type (SCCOHT) are a rare histotype. Gene mutations, copy number amplifications, methylation and other epigenetic silencing are noted against histotypes.

Almost all HGSOC have a somatic *TP53* mutation and p53 immunohistochemistry is a surrogate marker for *TP53* mutation in these tumors (Bell, 2011; Cole et al., 2016; Köbel and Kang, 2021). *TP53* mutations are also observed in MOC (Köbel and Kang, 2021), ovarian carcinosarcomas (Trento et al., 2020) and less frequently in OCCC (Parra-Herran et al., 2019). *BRCA1* and *BRCA2* mutations occur in HGSOC, including in the germline of affected patients (Alsop et al., 2012), and rarely in patients with OCCC (De Pauw et al., 2021). *ARID1A* and *ARID1B* encode members of the SWI/SNF (SWItch/Sucrose Non-Fermentable) ATP-dependent chromatin remodeling complex important for interaction of this complex with DNA, both genes being mutated in OCCC and endometrioid ovarian cancers (McCluggage and Stewart, 2021). The SWI/SNF complex is also disrupted in the very rare SCCOHT with mutation of *SMARCA4* and epigenetic silencing of *SMARCA2* that encode catalytic subunits important for nucleosome sliding and eviction (Jelinic et al., 2016; Xue et al., 2021). OCCC and endometrioid carcinomas also have in common a disrupted PTEN-PI3K pathway with mutations observed in *PTEN* and *PIK3CA*, as well as mutations in *CTNNB1* (Kuo et al., 2009; Hollis et al., 2020). The RAS/MAPK pathway has been implicated in LGSOC, with mutations identified in *KRAS*, *NRAS,* and *BRAF* (Moujaber et al., 2021). Mutations in the RAS/MAPK pathway are also observed in EnOC (Hollis et al., 2020), OCCC (Kim et al., 2018), and MOC (Cheasley et al., 2021). Adult ovarian GCTs are characterized predominantly by mutation of *FOXL2*, and Sertoli-Leydig Cell Tumors (SLCTs) harbor mutations in *DICER1* (Fuller et al., 2017; De Paolis et al., 2021). Epithelial and stromal cell ovarian cancer histotypes and associated genes known to be mutated are summarized in Figure 1.

In addition to mutations, methylation of *BRCA1* is also observed in HGSOC, as is amplification of *CCNE1* (Bell, 2011; Patch et al., 2015). Along with defects in *BRCA1* and *BRCA2*, other genes that function in homologous recombination repair (HRR) are mutated in ovarian cancer, albeit at lower frequencies, including *RAD51C*, *RAD51D*, *BRIP1*, *PALB2,* and *ATM* (Pennington et al., 2014). Defects in HRR can lead to the presence of “genomic scars” caused by the cancer cell’s inability to accurately repair sites of double strand breaks (DSBs). These include extensive loss of heterozygosity (LOH), large scale transitions (LST) and telomeric allelic imbalance (TAI) (Watkins et al., 2014; Chao et al., 2018; Nguyen et al., 2020). Tumors with defects in HRR are responsive to poly adenosine diphosphate-ribose polymerase inhibitors (PARPis), functioning in a synthetic lethal manner to inhibit repair of single strand breaks *via* the base excision repair pathway (Dedes et al., 2011). As a predictive DNA marker of defective HRR, genomic scars are helpful, but not all tumors with a genomic scar will respond to a PARPi. Reasons for this include reversion of a *BRCA* mutation, occurrence of a secondary mutation that restores wild-type function or changes in methylation patterns of an HRR gene that in effect functionally restores HRR but the genomic scar remains (Patch et al., 2015). Functional analyses, alongside molecular assays, are required to confirm the predicted response of women with ovarian cancer to PARPis such as the FDA-approved drugs olaparib, niraparib and rucaparib (Dickson et al., 2021; Lee et al., 2021; Valabrega et al., 2021).

Predicting which women will respond to a PARPi is particularly important given the unprecedented improvement seen in Progression Free Survival (PFS) and Overall Survival (OS) for subsets of women administered these drugs, including as maintenance therapy (Audeh et al., 2010; Ledermann et al., 2012; Ledermann J. et al., 2014; Moore et al., 2018). Ovarian cancers with germline or somatic mutation of *BRCA1* or *BRCA2*, mutation of *RAD51C* or *RAD51D*, methylation of *BRCA1,* or high LOH have all been reported to respond to PARP inhibition (Audeh et al., 2010; Wang et al., 2012; Ledermann J. et al., 2014; Kondrashova et al., 2017; Swisher et al., 2017; Moore et al., 2018). Furthermore, loss of *RAD51C* methylation has recently been implicated as a mechanism of PARPi resistance (Nesic et al., 2021). The combination of molecular testing and functional analyses conducted in robust tumor or ascites models has the power to strongly predict whether a woman is likely to respond to a PARPi at each stage of her disease progression.

Unlike for PARPis, there are no clinically approved biomarkers to predict responses of women with ovarian cancer to the anti-angiogenic bevacizumab, although numerous studies have focused on this area (Buechel et al., 2021; Gao et al., 2021; Hsu et al., 2021). Given the advances seen in PFS and OS of some women receiving this monoclonal antibody that targets vascular endothelial growth factor (VEGF), development of functional assays to predict the likelihood that a woman will respond to bevacizumab would represent a major advance (Burger et al., 2011; Perren et al., 2011; Oza et al., 2015).

The majority of women with HGSOC will respond initially to carboplatin, although some tumors display innate platinum resistance (Davis et al., 2014). There are no robust markers to predict response to platinum drugs, including through the development of acquired chemoresistance, although defects in HRR in primary tumors have been associated with a more favorable response (Konstantinopoulos et al., 2010; Muggia and Safra, 2014). The use of platinum drugs in histotypes other than HGSOC must be questioned given frequent low response rates. Only 11–27% of OCCC respond initially to platinum therapy, dropping to only 1-2% response rates in recurrent disease (Sugiyama et al., 2000; Mabuchi et al., 2016; Tan et al., 2019). LGSOC also display a poor response to platinum drugs; however, the presence of activating mutations in mitogen-activated protein kinase (MAPK) pathway genes *KRAS*, *BRAF,* and *NRAS* has seen favorable responses in LGSOC with the MEK inhibitor trametinib (reviewed in Moujaber et al. (2021)).

The mutations and genomic variations described above offer opportunities to develop new molecular-based therapeutic strategies to treat ovarian cancer subtypes. Some molecular events, such as those described in the HRR pathway, are already being targeted clinically by FDA-approved PARPis. For both discovery science and translational approaches to predicting which women are likely to benefit from which therapies, robust models are needed that expand upon traditional 2D cell culture and pre-clinical models, and include both molecular profiling and functional analyses. In this review, we discuss methods of 3D modelling that are either currently being employed in ovarian cancer cell lines, primary or metastatic tumor tissue and ascites, or have the potential to be used into the future for these purposes.

## 2 The Microenvironment–Considerations When Modelling Ovarian Cancer

### 2.1 Sites of Origin of Ovarian Cancer

There are numerous factors to examine when considering the microenvironment that supports the initiation, development and metastasis of ovarian cancer, not least being the very first microenvironment of these malignancies and that is the site of origin of the initial lesion. Many, perhaps most, HGSOC originate in the fallopian tube as Serous Tubal Intraepithelial Carcinomas (STICs), shedding onto the surface of the ovary and establishing a tumor (Crum et al., 2007; Karst et al., 2011; Perets and Drapkin, 2016; Zhang et al., 2019). This discovery has led to the generation of important models of non-cancerous fallopian tube epithelial cells transformed with c-Myc, H-Ras^V12^ or SV40 large T antigen (SV40 TAg) (Karst et al., 2011; Perets and Drapkin, 2016). These models complement those of normal ovarian surface epithelial (OSE) cells immortalized with factors including SV40 Tag, human telomerase (hTERT) and HPV-E6/E7 (Tsao et al., 1995; Davies et al., 2003; Kalli et al., 2004; Shin et al., 2018). OCCC and EnOC have been associated with endometriosis (Samartzis et al., 2020). While MOC has previously been reported as originating from metastatic deposits of primary tumors of the colon, stomach, pancreas and uterus, evidence now shows that these tumors do in fact arise in the ovary following a progression model commencing with benign and borderline precursor lesions (Ledermann J. A. et al., 2014; Cheasley et al., 2019). While constituting ∼10% of all ovarian cancers, tumors arising in ovarian stromal cells have different considerations. In contrast to epithelial cell tumors, the sex cord stromal tumor GCT arise in granulosa cells that produce estrogen (Jamieson and Fuller, 2012). Knowledge of the sites of origin of ovarian tumors is imperative to ensure selection of models that best address both research questions and translational approaches.

### 2.2 The Ovarian Cancer Microenvironment and Metastatic Spread

The microenvironment of an ovarian cancer consists of both tumor and non-tumor cells, including immune cells. Patient-derived organoids or tumoroids retain cellular heterogeneity and immune cells, thus are able to more strongly recapitulate a three-dimensional (3D) tumor microenvironment *ex vivo* compared to homogeneous cell lines (Hill et al., 2018; Kopper et al., 2019). Ovarian tumoroid cultures have been established from both ascitic fluid and primary tumors, to date primarily from the more common HGSOC but also from LGSOC, MOC, OCCC, and EnOC (Hill et al., 2018; De Witte et al., 2020; Nanki et al., 2020). These models show great promise for conducting *ex vivo* drug assays to predict therapeutic response in the women from which they were established (De Witte et al., 2020; Maenhoudt et al., 2020; Nanki et al., 2020; Gorski et al., 2021).

Given the location of ovaries, there are no anatomical barriers preventing metastasis to organs in the pelvic cavity including the uterus, bladder, rectum, and small intestine, as well as beyond the peritoneal cavity to organs such as the liver and lung (Motohara et al., 2019). Ovarian cancer cells detach from the primary tumor and are attracted to adipose-rich omental tissue. They disseminate by forming aggregates of multicellular spheroids that float in malignant ascitic fluid alongside fibroblasts, adipocytes, mesothelial, endothelial and inflammatory cells, as well as cell-free DNA, before “seeding” onto new microenvironments and establishing metastatic deposits (Ford et al., 2020). Given that many commercially available ovarian cancer cell lines are in fact established from ascites rather than primary tumors (Table 1, Supplementary Tables S1,S2) consideration should be given to including these cell types when establishing three dimensional models of ovarian cancer. This is especially relevant for the study of ovarian cancer cell metastasis. An example of this is the organotypic model used by Ford and colleagues to determine the ability of ovarian cancer cell lines undergoing depletion of genes of interest to metastasize and adhere to omental-type tissue (Henry et al., 2017).

**TABLE 1:** Ovarian cancer cell line origin, *in vivo* growth and classifications.

Cell line	OvCa Histotype	Specimen site	Growth *in vivo* in mice	Commercial availability	References
CaOV-3	HGSOC^a^ ^,^ ^b^ ^,^ ^d^	Ovary tumor	Yes: IP; No: SC, IB	ATCC	Buick et al. (1985) ^f^; Hernandez et al. (2016) ^g^
CaOV-4	HGSOC^a^ ^,^ ^c^ ^,^ ^d^	Fallopian tube metastasis	Yes: SC, IP, IB	ATCC	Hernandez et al. (2016) ^g^
COV318	HGSOC^a^ ^,^ ^d^ ^,^ ^e^	Ascites	No: SC, IP	ECACC	van den Berg-Bakker et al. (1993) ^f^; De Haven Brandon et al. (2020) ^g^
COV362**	HGSOC^a^ ^,^ ^d^	Pleural effusion	Yes: IP, forms ascites and IB; No: SC	ECACC	van den Berg-Bakker et al. (1993) ^f^; De Haven Brandon et al. (2020) ^g^
KURAMOCHI	HGSOC^a^ ^,^ ^b^ ^,^ ^d^	Ascites	Yes: SC; No: IP, IB	JCRB	Motoyama (1981) ^f^; De Haven Brandon et al. (2020) ^g^
OAW28	HGSOC^a^ ^,^ ^d^	Ascites	Unknown	ECACC	Wilson et al. (1996)
OV202	HGSOC	Primary tumor	Unknown	No	Conover et al. (1998) ^f^
OVCAR-3	HGSOC^a^ ^,^ ^b^ ^,^ ^d^ ^,^ ^e^	Ascites	Yes: SC, IP	ATCC	Hamilton et al. (1983) ^f^; Hernandez et al. (2016) ^g^, De Haven Brandon et al. (2020) ^g^
OVCAR-4	HGSOC^a^ ^,^ ^d^	Ascites	Yes: SC, IP; No: IB	MERCK Millipore	Pirker et al. (1985) ^f^; Hernandez et al. (2016) ^g^; De Haven Brandon et al. (2020) ^g^
OVKATE	HGSOC^a^ ^,^ ^d^ ^,^ ^e^	Solid metastasis	Yes: SC, IP	JCRB	Yanagibashi et al. (1997) ^f^ ^,^ ^g^; Mitra et al. (2015b) ^g^
OVSAHO	HGSOC^a^ ^,^ ^d^ ^,^ ^e^	Solid metastasis	Yes: SC; Yes: IP, forms ascites	JCRB	Yanagibashi et al. (1997) ^f^ ^,^ ^g^; De Haven Brandon et al. (2020) ^g^
PEO1	HGSOC^c^	Ascites	No	ECACC	Langdon et al. (1988) ^f^; Hernandez et al. (2016) ^g^
PEO4	HGSOC^c^	Ascites	No	ECACC	Langdon et al. (1988) ^f^, Hernandez et al. (2016) ^g^
UWB1.289	HGSOC^c^	Ovary tumor	No: SC, IP	ATCC	DelloRusso et al. (2007) ^f^; Mitra et al. (2015b) ^g^
UWB1.289 + BRCA1	HGSOC^c^	Ovary tumor	Unknown	ATCC	DelloRusso et al. (2007) ^f^
A2780*	EnOC^a^ ^,^ ^b^ ^,^ ^c^ ^,^ ^d^	Tumor tissue	Yes: SC and IP, forms ascites	ECACC	Behrens et al. (1987) ^f^; Hernandez et al. (2016) ^g^
TOV-112D	EnOC^b^ ^,^ ^d^ ^,^ ^e^	Ovary tumor	Yes: IP; No: SC	ATCC	Provencher et al. (2000) ^f^; Hernandez et al. (2016) ^g^
OVISE	OCCC^a^ ^,^ ^b^ ^,^ ^d^ ^,^ ^e^	Solid pelvic metastasis	Yes: SC; No: IP	JCRB	Gorai et al. (1995) ^f^; Yanagibashi et al. (1997) ^f^ ^,^ ^g^
OVMANA	OCCC^a^ ^,^ ^b^ ^,^ ^d^ ^,^ ^e^	Primary tumor	Yes: SC; No: IP	JCRB	Yanagibashi et al. (1997) ^f^ ^,^ ^g^
OVTOKO	OCCC^a^ ^,^ ^b^ ^,^ ^d^ ^,^ ^e^	Solid splenic metastasis	Yes, SC; Yes: IP	JCRB	Gorai et al. (1995) ^f^; Yanagibashi et al. (1997) ^f^ ^,^ ^g^
RMG-I	OCCC^a^ ^,^ ^d^ ^,^ ^e^	Ascites	Yes: SC	JCRB	Nozawa et al. (1991) ^f^; Kashiyama et al. (2014) ^g^
TOV-21G	OCCC^a^ ^,^ ^b^ ^,^ ^d^ ^,^ ^e^	Ovary tumor	Yes: SC	ATCC	Provencher et al. (2000) ^f^; Kashiyama et al. (2014) ^g^
MCAS	MOC^b^ ^,^ ^e^	NS	Yes: SC	JCRB	Kidera et al. (1985) ^f^; Sato et al. (2012) ^g^
RMUG-S	MOC^d^ ^,^ ^e^	Ascites	Yes: SC, IP	JCRB	Sakayori et al. (1990) ^f^; Sato et al. (2012) ^g^; Matsuo et al. (2011) ^g^
KGN	GCT	Tumor tissue	Unknown	RIKEN BRC	Nishi et al. (2001) ^f^
COV434***	SCCOHT	Primary tumor	Unknown	No	van den Berg-Bakker et al. (1993) ^f^; Karnezis et al. (2021)

Note: Cell lines identified with >50 publications *via* PUBMED, on 10/12/2021.

OvCa, Ovarian Cancer; NS, Not specified; ATCC, American Type Culture Collection; JCRB, Japanese Cancer Research Resources Bank; ECACC, European Collection of Authenticated Cell Cultures; RIKEN BRC, RIKEN, BioResource Center Cell Bank; SC, subcutaneous; IP, intraperitoneal; IB, intrabursal.

* Originally classified HGSOC,

** Originally classified EnOC,

*** Originally classified as a GCT (Granulosa Cell Tumor),

Recent classification of histotypes.

a Domcke et al. (2013),

b Anglesio et al. (2013),

c Beauford et al. (2014),

d Barnes et al. (2021),

e Papp et al. (2018).

f Original histotype reference,

g *in vivo* tumour growth in mice reference.

EnOC, Endometrioid Ovarian Cancer; OCCC, Ovarian Clear Cell Carcinoma; MOC, Mucinous Ovarian Cancer; HGSOC, High Grade Serous Ovarian Cancer; SCCOHT, Small Cell Carcinoma of the Ovary, Hypercalcemic Type.

## 3 Ovarian Cancer Cell Line Models

To date, the majority of *in vitro* studies in ovarian cancer have relied on the use of 2D cell culture of immortalized cell lines derived from primary ovarian cancers, pleural effusion, ascitic fluid from the peritoneal cavity or a distant metastatic site. Many cell lines have been well characterized morphologically and molecularly and, when able to be tested, maintain unique features of their derivative sample. Several studies have attempted to determine “the best” ovarian cancer cell line models for investigators to use for both fundamental discovery science and translational projects (Anglesio et al., 2013; Domcke et al., 2013; Beaufort et al., 2014; Papp et al., 2018; Barnes et al., 2021). Comparison of the molecular profiles of ovarian cancer cell lines with that of primary tumors has led to the histotype reclassification of a number of frequently used ovarian cancer cell lines, including SK-OV-3 and A2780. Still, there remains conflicting reports in the field as to the accuracy of some ovarian cancer cell lines. We have summarized the current state of knowledge of site and histotype origin of a group of ovarian cancer cell line models, as well as models of normal cells representing sites of origin (Table 1, Supplementary Tables S1–S3).

With the exception of the PEO series of HGSOC, few ovarian cancer cell line models allow insight into the development and progression of ovarian cancer (Langdon et al., 1988). The PEO1 drug sensitive cell line has the pathogenic *BRCA2* mutation, c.5193C > G, derived after initial treatment with cisplatin, 5-Fluorouracil (5-FU) and chlorambucil. The PEO4 cell line represents malignant cells after the patient developed chemoresistance, having a secondary *BRCA2* reversion mutation which restores wild-type BRCA2 function. The PEO6 cell line was collected from the same patient before death (Langdon et al., 1988). Other ovarian cancer cell lines have been made resistant to cisplatin *in vitro*, including A2870/A2780CisR (Behrens et al., 1987), TYK-nu/TYK-nu.CP-r (Yoshiya et al., 1989) and CaOV3/CaOV3CisR (Joshi et al., 2021). The A2780/A2780VeliR lines were made resistant *in vitro* to the PARPi veliparib (Dickson et al., 2021). Still, often phenotypes such as drug response observed *in vitro* have been unable to mirror *in vivo* models, likely due to factors missing in the tumor microenvironment that are absent from homogeneous cell lines cultures such as stromal and immune cells (Beaufort et al., 2014).

Ovarian cancer cell lines show variable ability to grow in nude mice when implanted either subcutaneously (SC), intraperitoneally (IP) or intrabursally (Hernandez et al., 2016). Further, while some cell lines grow well in both SC and IP locations, others show a strong propensity to grow in one location only, suggesting a preference for a particular microenvironment. Ovarian cancer cell lines demonstrated to grow in mice are noted in Table 1 and Supplementary Tables S1,S2.

## 4 Mouse Models of Ovarian Cancer

Animal models continue to be the most physiologically relevant pre-clinical models to study disease pathogenesis and drug response, encompassing a whole-body system, including immune system, tumor microenvironment and vascularization. A number of non-mammalian models including fruit flies, the African clawed frog (*Xenopus*) and the laying hen, have been utilized for the study of ovarian cancer development (reviewed by (Johnson and Giles, 2013; Rosales-Nieves and González-Reyes, 2014; Bernardo et al., 2015; Tudrej et al., 2019)). The most widely used mammalian model is the mouse (*Mus musculus*), sharing 85 percent protein-encoding gene homology with humans (Makałowski et al., 1996), although concerns with translatability of disease mechanisms and drug responses between species remain. Further, the natural occurrence of ovarian cancer is low in the aging mouse, with rapid progression times contrasting with the development of human ovarian cancer (Sale and Orsulic, 2006). Nevertheless, genetically engineered mouse (GEM) models, syngeneic and patient-derived xenograft (PDX) models have enabled a greater understanding of ovarian cancer development and treatment responses.

### 4.1 GEM and Syngeneic Models

GEM models have enabled specific gene knockout to be modelled in a whole-body system, contributing to the understanding of individual and combinations of genes commonly mutated in ovarian cancer. Conditional knockout mice, using the Cre-lox system for cell type specificity, have been used to reproduce oncogenic mutations and HR defects to study ovarian cancer development and responses to clinically relevant treatments such as platin-based drugs and PARPis (Szabova et al., 2012; Perets et al., 2013; Szabova et al., 2014; Zhai et al., 2017). Extensive overviews of GEM models of ovarian cancer have been published in recent years, highlighting comparisons and translatability to the human condition (Stuckelberger and Drapkin, 2018; Maniati et al., 2020; Zakarya et al., 2020). Syngeneic mouse models transplant mouse cell lines into a recipient from the same genetic background, enabling the study of immune response, immunotherapies and tumor vascularization (Zhang et al., 2002; Yu et al., 2018). The murine ID8 ovarian cancer cell line (C57Bl/6 background) (Roby et al., 2000), has been used for a number of syngeneic mouse models, achieving primary ovarian tumors and ascites within 90 days (Greenaway et al., 2008). This model has also been used to study metastasis and immune infiltrates at the trocar site, where an incision is made into the abdomen for laparoscopic surgery (Wilkinson-Ryan et al., 2019). Injection with M0505 cells (derived from spontaneously transformed OSE of FVB/N mice) resulted in Pax8+ tumors with similar histology to human HGSOC (McCloskey et al., 2014). Generation of multiple fallopian tube epithelial cell lines with combinations of common mutations in HGSOC (*Tp53*, *Brca1*, *Brca2*, *Ccne1*, *Akt2*, *Brd4*, *Smarca4*, *Kras*, *Myc*, *Nf1*, and *Pten*) using CRISPR/Cas9 recapitulated histopathological and clinical features observed in HGSOC patients, such as ascites and peritoneal metastases (Iyer et al., 2021). Overall, GEM and syngeneic models have proven their value for discovery studies and research into the origin of ovarian cancer.

### 4.2 Patient-Derived Xenograft Models

PDX models are the most useful *in vivo* model for testing response to targeted therapies of primary tumors attributed to their unique molecular profiles, to enable a precision medicine approach. The major advantage of using PDX models is the ability to reproduce histology of the human tumor (Colombo et al., 2015), although alterations in steroid hormone receptors and immune response genes have been reported, irrespective of the maintained mutational profile (Dong et al., 2016). Another advantage of PDX models is that they bypass the *in vitro* culture of tumor cells that may inadvertently drive phenotype divergence from the original tumor (Siolas and Hannon, 2013).

Limitations of primary tumor tissue implanted heterotypically into immunodeficient mice include the inability to recapitulate immune responses, site-specific tumor microenvironment interactions and lastly, that the tumor may not metastasize (Jin et al., 2010). The general methodology of producing PDX models requires multiple *in vivo* passages, leading to extended model creation times (Morton and Houghton, 2007). The reported engraftment rate can be variable and heavily influenced by ovarian cancer histotype; treatment history; stage and site of malignancy, with higher engraftment rates observed in non-epithelial histotypes (Wu et al., 2019). Platinum resistance has also been found to predict PDX engraftment success, with successful engraftment correlating with shorter PFS and OS of the derivative patient (Heo et al., 2017).

An extensive review of ovarian cancer PDX models by Scott et al. (2013), highlighted a number of gaps in the field related to variations in methodologies, genetic stability over multiple generations, representation of few ovarian cancer histotypes and the limitation of using immunocompromised mice as hosts. More recent developments have addressed some of these concerns, with higher rates of successful engraftment and propagation of rarer ovarian cancer histotypes such as LGSOC (De Thaye et al., 2020) and MOC (Ricci et al., 2020) as well as evaluation of drug responses for homologous recombination deficient (HRD) mutated ovarian cancers (George et al., 2017) and enabling the evaluation of immunotherapies through the use of humanized mouse models (Odunsi et al., 2020). Furthermore, the ability to label tumor cells with luciferin prior to transplantation has enabled tumor growth tracking *via* bioluminescence imaging (Liu et al., 2017).

## 5 3D *in vitro* Ovarian Cancer Models

Two-dimensional (2D) growth of cancer cells as monolayers may fail to recapitulate aspects of the derivative cell behavior and morphology. Differential drug responses in 2D *versus* 3D cultures have been observed in many *in vitro* models of cancer, including ovarian cancers. Previously, high costs of materials, significant manual labor and low levels of reproducibility and matrix tunability rendered 3D culture models less favorable to 2D, irrespective of their higher physiological relevance (Jensen and Teng, 2020). Recent technological advances have enabled higher degrees of control over the creation of 3D cell cultures in areas including matrix stiffness and composition, spatial orientation and creation in an automated and high-throughput manner.

A number of different methodologies and techniques are being used in order to more efficiently create 3D cell culture models of cancer, including ovarian cancers. While there is still no “perfect” 3D *in vitro* model that can replace *in vivo* preclinical models, methodological advances are moving the field towards more accurate representations of human tumors, including in regards to drug response. Further, efforts towards creation of high-throughput *in vitro* and *ex vivo* models for drug screening have been a focus in recent years, and will eventually replace 2D cultures. An overview of techniques used to create 3D *in vitro* ovarian cancer models and their considerations is summarized in Table 2.

**TABLE 2:** Advantages and disadvantages of common 3D *in vitro* models of ovarian cancer.

Model type	Technique	Advantages	Disadvantages
Scaffold-free	Liquid overlay—Flat-bottom plates	Fast spheroid generation	Heterogenous spheroids
			No cell-ECM interactions
	Liquid overlay—Round-bottom plates	Fast spheroid generation	May require Matrigel for cell-cell adhesion
		May replicate necrotic core	No cell-ECM interactions
	Hanging drop	High homogeneity	Difficulties with media change, drug addition
		Fast spheroid generation	No cell-ECM interactions
Scaffold-based—Natural hydrogels	Matrigel	High biocompatibility	Not human derived
		Integrin interactions	Limited control of mechanical properties
		Commercially available	Temperature dependent stability
		Mimics basement membrane ECM	Batch-to-batch variation
		Enables organoid propagation	
	Collagen-I	High biocompatibility	Not human derived
		Enhances mesenchymal traits	Limited control of mechanical properties
		Variety of sources (animal, marine)	
	Alginate	High biocompatibility	Stiffness modulated by multivalent cations (possible cytotoxicity)
		Low immunogenicity	No cell-ECM interaction
		Can be combined with other biomaterials	
	Agarose and Agar	High biocompatibility	Innately inert for cell adhesion studies
Scaffold-based—Synthetic hydrogels	Polyethylene glycol (PEG)	Tunable stiffness	Requires biofunctionalization
		Low batch-to-batch variation	
		Able to be used as bioink for bioprinting	
	Gelatin methacryloyl (GelMA)	High biocompatibility	UV photocrosslinking may cause DNA damage
		Innate RGD and MMP cleavability	
	Peptide-based e.g. RADA16-I	Defined nanofibers	Low mechanical strength
		Highly engineerable	
		Self-assembling	
Decellularized ECM		High biocompatibility	Limited control of mechanical properties
		Retention of native ECM and growth factors	Donor heterogeneity
Organotypic omental mesothelial model		Modelling metastasis to omentum	Reliance on primary cells (when used)
		Organotypic co-culture	No vasculature
Organoids		Maintenance of patient mutational profile and tumor histology	No vasculature
		Can be biobanked	Loss of stromal and immune cells in longer-term culture
		Can predict patient responses	Varied success rates
		CRISPR-editable	
3D Bioprinting	Droplet	High-throughput	High equipment cost
		High precision	Limited compatible bioinks
	Extrusion	Compatible with multiple ECM types	Low-throughput
			Potential for cell stress during extrusion process
			Low precision
Bioreactors	Rotating wall vessel	Mimic microgravity and transcoelomic metastases	Only spheroid culture
	Orbital shakers	Spheroid formation studies	Only spheroid culture
		Maintenance of patient-derived explants	
	Compressive stress	Hydrostatic compression stress	Not commercially available
	Tumor-on-a-chip	Model shear stress on EMT	Short-term culture
		Able to control drug or nutrient gradients	Potential variation between in-house fabricated devices

Note: ECM, extracellular matrix; MMP, matrix metalloproteinase; UV, ultraviolet; EMT, epithelial-mesenchymal transition.

### 5.1 Scaffold-Free Models

#### 5.1.1 Liquid Overlay Techniques

Liquid overlay techniques, such as the use of ultra-low attachment (ULA) plates or low-attachment coatings enable spheroid formation attributed to the hydrophilic properties of the neutrally charged polystyrene plastic or polymer coating, causing cells to adhere to each other rather than on a 2D surface. By preventing attachment to a surface, use of low attachment plates and coatings present a cost-effective and timely method for spheroid formation or maintenance of existing spheroid structures. Two methods have been used extensively for ovarian cancer spheroid culture to identify mechanisms of progression and various stages of disease, from primary tumor modelling to the generation of spheroids of metastatic ascites. The attachment and disaggregation of these spheroids on top of an ECM or in an immunodeficient mouse also allows assessment of the metastatic potential of the cancer.

##### 5.1.1.1 Flat-Bottomed Ultra-Low Attachment Plates and Low Attachment Coatings

**FIGURE 2:**
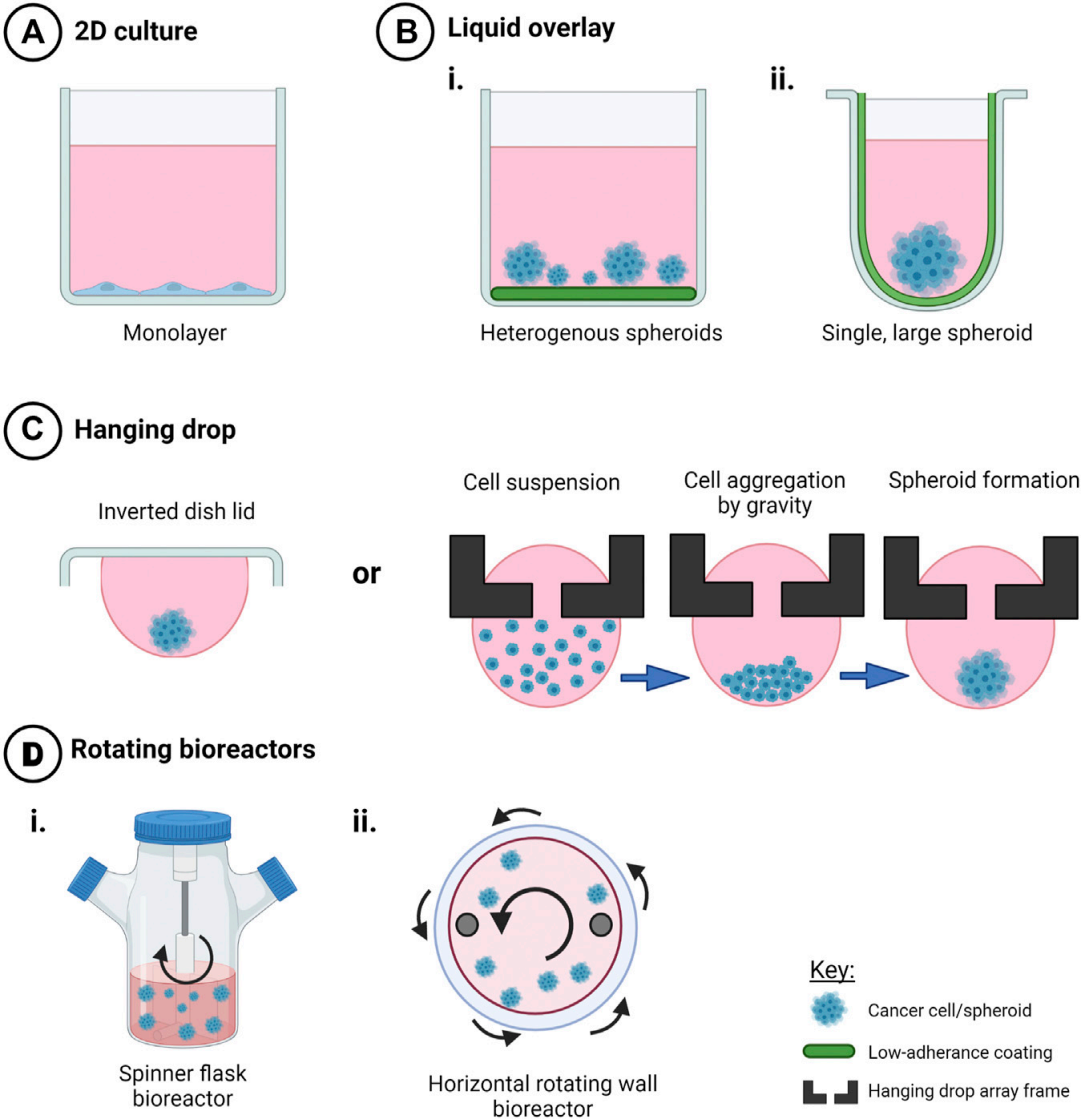
Techniques to create scaffold-free 3D *in vitro* cancer models. Creation of 3D cell models in the absence of scaffolds promotes cell-cell interactions in three dimensions that mediate cell behavior and drug response when compared to **(A)** 2D monolayers. Use of **(B)** liquid overlay techniques with i) flat or ii) round-bottomed ULA plates **(C)**, hanging drop techniques and **(D)** rotating bioreactors such as i) spinner flasks and ii) horizontal rotating vessels have been used as time and cost-effective spheroid creation methods or to investigate drug response and other factors that my influence ovarian cancer progression, such as fluid shear stress and hypoxia. Created with Biorender.com.

The use of flat-bottomed ULA plates (Figure 2) enables heterogenous multicellular aggregate formation from cell suspensions of adherent cells and can be used for short-term maintenance of primary ascites-derived spheroids. This method is often combined with secondary metastatic invasion assays involving the transfer of spheroids to regular tissue culture plastic plates or onto an extracellular matrix (ECM).

Culture of ovarian cancer cell lines in ULA plates as “ascitic” spheroids has been used as a model to investigate the efficacy of an oncolytic virus-based therapeutic on ovarian cancer metastasis (Tong et al., 2015). Patient-derived ovarian cancer ascites cells, when maintained on ULA plates, demonstrated epithelial-mesenchymal transition (EMT) during spheroid formation (Rafehi et al., 2016). Patient-derived solid-tumor and ascites-derived ovarian cancer cell lines both form spheroids similar to those found in patient ascites when grown on ULA plates, and were shown to be self-renewing through serial passaging over a 6-month period (Yamawaki et al., 2021). Patient-derived spheroids were also more tumorigenic in immunodeficient mice, more stem-like and more invasive than their parental cell line (Liao et al., 2014). A direct comparison of cisplatin and paclitaxel sensitive and resistant A2780 cells grown as 2D monolayers *versus* 3D aggregates formed in ULA plates, identified gene expression changes attributed to conformation that may lead to drug resistance (Nowacka et al., 2021).

Poly(2-hydroxyethyl methacrylate), known as poly-HEMA, is a non-ionic polymer coating that discourages the formation of ECM and creates a low-adhesion environment that favors spheroid formation (Ivascu and Kubbies, 2006). A poly-HEMA coating in culture flasks has been used for the production of 3D heterotypic models of normal ovary and to study early ovarian cancer development (Lawrenson et al., 2012). Ovarian cancer cell lines grown under poly-HEMA conditions in the presence of activated platelet releasate, formed spheroids faster and were more resistant to the chemotherapeutic agents cisplatin, carboplatin and paclitaxel (Casagrande et al., 2021).

While flat-bottomed, ULA plates and non-adherent coatings present a cost-effective method for production and maintenance of ovarian cancer spheroids, these methods result in heterogenous spheroid morphologies which may impact on downstream analysis, reproducibility and drug response. As such, we suggest that this method is best suited for investigations into drivers of spontaneous spheroid formation.

##### 5.1.1.2 Round-Bottomed ULA Plates

The use of round-bottomed ULA plates (Figure 2) facilitates single spheroid formation by gravity, and is often supplemented with ∼2% Matrigel to fast track cell aggregations over periods of time less than 72 h. This method allows high consistency between replicates from a cell line or patient sample, as well as facilitating the ability to section and morphologically analyze biologically-relevant structures. Formation of single large spheroids (>500 μm diameter) enables the formation of a hypoxic core, and drug, oxygen and metabolite gradients that mimic solid tumor physiology (Vinci et al., 2012; Heredia-Soto et al., 2018).

The use of round-bottomed, ULA plates have assisted in the identification of Nectin-4 as essential for adhesion events in early spheroid formation of HGSOC cell lines, and as potential targets to improve chemotherapeutic sensitivity (Boylan et al., 2020). Pre-formed OVCAR-8 spheroids grown in round-bottomed ULA plates and further embedded as single spheroids in Matrigel, developed vascularization after subcutaneous transplantation into athymic nude mice, enabling the evaluation of therapeutics such as the anti-angiogenic bevacizumab and the nano-drug Doxil^®^ (Singh et al., 2020).

This method of spheroid formation has been utilized for speed, reproducibility and commercial availability, although spheroid morphology and overall size may differ due to intrinsic cell line-related characteristics. In a comparison of hanging drop arrays, liquid overlay on ULA plates and liquid overlay on ULA plates with a nutator device to produce three dimensional agitation, both the hanging drop and ULA plates with agitation demonstrated higher cellular compaction, higher ECM content and increased resistance to cisplatin compared with cultures on liquid overlaid ULA plates only (Raghavan et al., 2016). Addition of agitation may improve the comparability to other scaffold-free spheroid formation techniques, although this is yet to be tested on primary ovarian cancer cells.

#### 5.1.2 Hanging Drop Techniques

Hanging drop cultures rely on surface tension and gravity to form homogenous, multicellular spheroid/aggregate cultures without the need for specialized equipment (Figure 2). Suspended cells in media are seeded either on the lid of a culture dish or in hang drop vessels, gather at the base of the droplet by gravity, aggregate by cell-cell integrin bridges and further mature by cell-mediated contraction to form compact spheroids within days (Sodek et al., 2009).

The hanging drop technique has been used in numerous studies investigating ovarian cancer morphology and phenotypes. The simplicity of the model has enabled it to act as a “low-stiffness” model as compared to traditional polystyrene plastic culture dishes (Mieulet et al., 2021). An advantage of this technique, particularly for low-volume primary tumor samples, is the formation of spheroids within days with high viability. Further, cell numbers as low as 10 cells per droplet will form spheroids that are uniform in both volume and circularity, amenable to many downstream analyses including high throughput analysis of drug responses (Raghavan et al., 2015).

The use of the hanging drop technique has led to the identification of mechanisms that promote ovarian cancer progression, chemoresistance and recurrence. A study of six ovarian cancer cell lines found a positive relationship between EMT status, spindle-like morphology and compactness of the formed spheroid, with more mesenchymal ovarian cancer cells exhibiting greater invasive and chemoresistant phenotypes (Sodek et al., 2009). Serial passaging of OVCAR-3 and ascites-derived spheroids in the hang drop system showed increasing stemness, proliferation, resistance to cisplatin and tumorigenicity *in vivo* with passage age (Ward Rashidi et al., 2019). Using a high-throughput 384-well hang drop array culture, increasing spheroid size and cell count was associated with resistance to cisplatin in A2780 and OVCAR-3 cells, which has impact particularly in ascitic spheroids escaping chemotherapeutic treatment (Raghavan et al., 2015). The stem-like changes and chemoresistance observed in this simple, multicellular spheroid model that would not be observed in traditional 2D cultures, emphasizes the importance of three-dimensional cell-cell interactions when modelling drug response. 3D heterotypic multicellular tumor spheroids generated by the hanging-drop technique using the cell lines HEY or SK-OV-3 in co-culture with the mouse fibroblast line NIH3T3, were used to identify off-target effects of drugs targeting cancer cells relative to neighboring stromal cells (Weydert et al., 2020).

While simple in design, using the hanging drop technique for spheroid creation has logistical issues with media replacements, drug addition, evaporation and downstream analysis of individual spheroids per hanging drop (Kunz-Schughart et al., 2004; Mehta et al., 2012). These models are therefore limited to short-term culture and require frequent attention. Improvements to the efficiency and reproducibility of this method include development of hanging drop arrays for use with liquid handling robotics and single cell seeding in nanoliter-sized wells in a microchip format (Raghavan et al., 2015; Ganguli et al., 2021). Creation of an open-source, 3D printable multi-purpose hanging drop “dripper” for use with standard tissue culture plates enables metastasis and migration assays as well as co-cultures of cells within the same plate (Zhao L. et al., 2019).

### 5.2 Scaffold-Based Hydrogel Models

**FIGURE 3:**
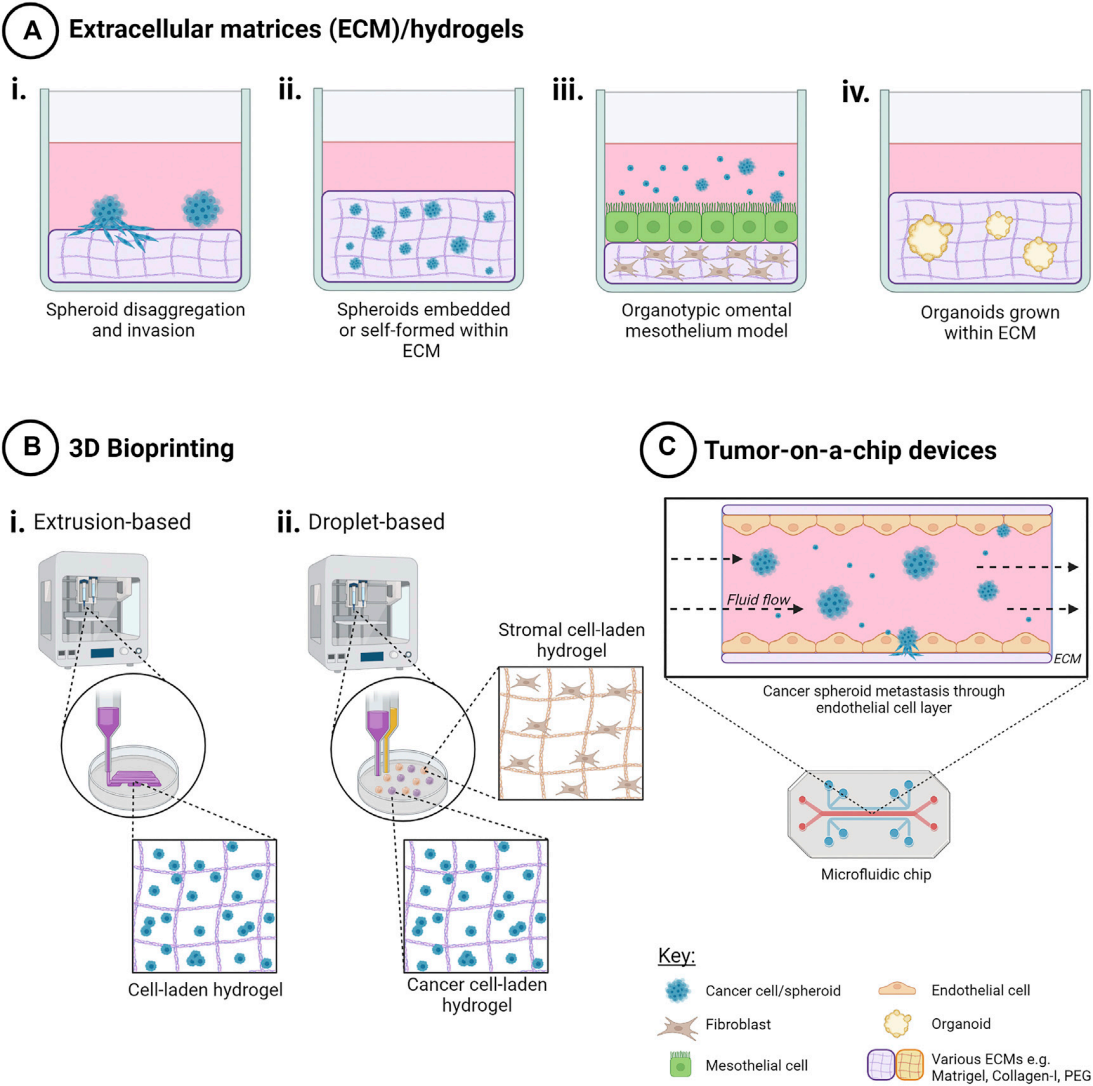
Techniques to create 3D *in vitro* cancer models using scaffolds. Addition of extracellular matrix (ECM) as scaffolds for 3D cell cultures enables both cell-cell and cell-ECM interactions for a more physiologically relevant 3D cancer cell model. Methods include **(A)** ECM/hydrogels with cancer cells i) on top of, or ii) encapsulated within an ECM, iii) organotypic omental co-culture model and iv) organoid propagation. **(B)** 3D bioprinting techniques such as i) extrusion-based bioprinting enables creation of 3D cell-laden models in hydrogels in a layer-by-layer manner, and ii) droplet-based bioprinting enabling high-throughput creation of 3D cell models in hydrogels with higher spatial control for more complex co-culture. **(C)** Tumor-on-a-chip microfluidic devices have been used to model the effects of fluid shear stress, as well as simulating nutrient, gas and drug gradients, for ascites metastasis modelling. Created with Biorender.com.

Inclusion of scaffolds in 3D multicellular *in vitro* models of ovarian cancer adds another level of model complexity, allowing for the recreation of the physical and mechanical tumor microenvironment that can influence ovarian cancer cell behavior. Several methodologies pertaining to the use of scaffolds relevant to ovarian cancer are described here and in Figure 3.

#### 5.2.1 Natural Scaffolds

Naturally derived ECM hydrogels such as Matrigel, collagens, alginate, and agarose have been favored in 3D cell culture models due to their history of high biocompatibility with various cell types, including ovarian cancer cell lines and tumor organoids, although they are limited in their mechanical tunability and composition consistency.

##### 5.2.1.1 Matrigel

Matrigel, derived from the murine Engelbreth-Holm-Swarm (EHS) sarcoma, has been used for over 40 years as a mimic for the basement membrane and a structural support for many cell types (Orkin et al., 1977). The major constituents of this ECM are laminins, collagen IV, entactin, and the heparin sulfate proteoglycan perlecan (Hughes et al., 2010), though ratios often differ by batch, raising the need for caution when interpreting results of cells cultured in Matrigel (Vukicevic et al., 1992). Matrigel or EHS matrix may also contain collagens I, XVIII, VI, and III, alongside growth factors and enzymes such as TGF-β, FGF, and matrix metalloproteinases (MMPs) (Hughes et al., 2010).

Matrigel has been widely used for *in vitro* ovarian cancer models as the ECM components overlap with those found *in vivo*. As a matrix, Matrigel has been utilized for the assessment of *in vitro* ovarian cancer cell invasion by cell penetration through Matrigel-coated transwell inserts (Woo et al., 2007; Fujisawa et al., 2012; Hallas-Potts et al., 2019). Metastatic outgrowth of ovarian cancer spheroids embedded within Matrigel has also been assessed (Sodek et al., 2008). Numerous ovarian cancer cell lines have been reported to require encapsulation within Matrigel or prior growth in Matrigel for successful seeding as *in vivo* tumors into athymic nude mice (Mullen et al., 1996). Growth of ovarian cancer cells on top of a Matrigel bed has been used to model seeding of metastatic ovarian cancer onto the peritoneum, resulting in ovarian cancer nodules in the absence of vascularization, and modelling chemoresistant metastases in the presence of a hypoxic core (Evans et al., 2011). At a 2-2.5% concentration in culture media, Matrigel has been utilized to promote efficient formation of dense, single spheroids of a number of cancer cell types, including ovarian, over 24 h in poly-HEMA treated, non-adherent, round-bottom plates for high-throughput toxicity studies (Ivascu and Kubbies, 2006). Further, Matrigel has been used as a scaffold to propagate primary cell types associated with ovarian cancer such as mesothelial cells and fibroblasts (Kenny et al., 2007), as well as to establish ovarian cancer tumoroids from patient biopsies that maintain key characteristics of the primary tumor (Maenhoudt and Vankelecom, 2021).

Aside from commercial availability and high biocompatibility with a wide range of cell types, there continues to be concerns regarding the use of Matrigel in a number of areas, including: batch-to-batch variation, potential for xenogeneic contaminants derived from mouse on human cells, inefficient cell retrieval requiring temperature reversal to 4°C, potential DNA/RNA contamination from the matrix, and matrix autofluorescence that reduces signal-to-noise ratio when imaging fluorescently stained cells *in situ* (Graf and Boppart, 2010; Hughes et al., 2010). The reported average modulus value of Matrigel was found to be approximately 450 Pa (Soofi et al., 2009), which remains below the average stiffness of human or mouse ovary or omentum (Alkhouli et al., 2013; Mckenzie et al., 2018; Hopkins et al., 2021). Further, the polymerization temperature for Matrigel of 22–37°C, mediated by entactin interaction with laminins and collagen IV, also limits its bioprintability in the absence of temperature-controlled environments or as a hybrid bioink (Fan et al., 2016).

Overall, Matrigel continues to be one of the most versatile ECMs for *in vitro* modelling of ovarian cancer cells. Synthetic alternatives are becoming more prominent but have yet to meet the standards of enabling the propagation of patient-derived spheroids and organoids as well as having mechanical properties to enable bio-printing for highly reproducible and high-throughput ovarian cancer model development. Until this time, Matrigel’s major advantages lie with its rich ECM composition for biocompatibility with a wide range of cell types, including for ovarian cancers.

##### 5.2.1.2 Collagens

Collagens are the most abundant ECM proteins in the body and are also the most widely used ECM for *in vitro* cell cultures due to high biocompatibility and commercial availability. Collagens provide structural support and facilitate cell adhesion, proliferation, differentiation and migration both *in vivo* and *in vitro*. Made up of 28 identified collagen types, the low antigenicity of collagen has enabled cross-species compatibility and in turn, availability from a variety of biological sources, including rat, bovine, porcine, and recombinant human (Lynn et al., 2004; Yang et al., 2004). Excessive macrofibrillar collagen-I produced by resident fibroblasts has been implicated in the promotion of cancer progression, metastatic and chemoresistant tumor microenvironments (Gurler et al., 2015). Crosslinking of collagen that increases stromal stiffness has also been implicated in chemoresistance (Sterzyńska et al., 2018). While there have been anti-cancer therapeutic strategies developed to enhance the efficiency of chemotherapy (Zhao Y. et al., 2019), collagen is abundant in both pathophysiological and normophysiological states and therefore, anti-collagen therapies require a targeted method of delivery (Xu et al., 2019).

Collagens, particularly collagen-I, have been used extensively in 3D ovarian cancer spheroid and organotypic cultures. OVCAR-5 spheroids plated on a collagen-I coating, were observed to readily disaggregate with increasing collagen concentration, compared to laminin or collagen-IV coatings, thus highlighting its properties as a promoter of cancer cell adhesion and invasion (Burleson et al., 2004). Rat-tail collagen-I has been used as the ECM to support primary mesothelial and fibroblast cells in an omental model of ovarian cancer metastasis (Kenny et al., 2007). Encapsulation of human ovarian cancer cell lines derived from primary tumors in collagen-I hydrogels showed enhanced mesenchymal traits, invasiveness and drug resistance (Liu et al., 2018). Microfibrillar collagen-VI, in addition to collagen-I, has also been reported to promote platinum resistance, cancer cell survival and HGSOC relapse both *in vivo* and *ex vivo* in collagen hydrogels (Pietilä et al., 2021). Collagen-XI has been implicated in tumor aggressiveness and poor clinical outcome of patients with ovarian cancer (Wu et al., 2014), although it has not been used as a scaffold for 3D ovarian cancer cultures. Marine collagen sources, such as from jellyfish species, have been found to have high amino acid similarity to collagen-I from mammals, showing similar *in vitro* cell behavior responses to traditional collagen-I matrices, and as such, are being investigated as a more sustainable collagen source (Paradiso et al., 2019).

##### 5.2.1.3 Alginate

Alginate is a natural polysaccharide derived from the brown algae *Phaeophycota*. Features such as high biocompatibility, biodegradability, low immunogenicity, and low cost have been the drivers behind its use in tissue engineering and drug delivery studies (Gombotz and Wee, 1998). Control of the degree of gelation, and in turn stiffness, can be modulated by multivalent cations including Ca^2+^, Fe^3+^, or Sr^2+^ for crosslinking. These cations can also mediate individual biofunctional properties such as cell attachment or absorption of serum proteins (Machida-Sano et al., 2009). However, proteins are unable to interact with the matrix, and as such ECM-cell signaling is lacking in alginate models and is considered a more synthetic ECM that can be biofunctionalized by the addition of peptides (Rowley et al., 1999). Further, certain cations may also induce cytotoxicity and may differ in stability.

While more studies have used alginate as a drug delivery system, alginate and alginate mixes, such as chitosan-alginate, have also been used as 3D hydrogel scaffolds for the enrichment of a cancer stem cell (CSC) phenotype in prostate, breast and hepatocellular carcinomas (Florczyk et al., 2016). Alginate encapsulation has been used as a method for *ex vivo* culture of murine ovary slices for the investigation of ovarian surface epithelial changes that may drive cancer development (King et al., 2011). Many reports have also combined alginate with other ECMs, both natural and synthetic, to create more biofunctionalized scaffolds to support a variety of ovarian cell types. Shin and colleagues combined alginate, for its biocompatible properties, with marine collagen and agarose to create a hydrogel that supported the growth of A2780 endometrioid ovarian cancer cells (Shin et al., 2016). A double network of alginate and polyethylene glycol (PEG)-based hydrogels was shown to increase doxorubicin resistance and CSC markers in SK-OV-3 cells compared to 2D cultures (Zhou et al., 2020). SK-OV-3 cells grown in these scaffolds were also reported to be more tumorigenic in a triple immunodeficient mouse model NCG (NOD-*Prkdc* ^*em26Cd52*^ *Il2rg* ^*em26Cd22*^/NjuCrl) (Zhou et al., 2020).

##### 5.2.1.4 Agarose and Agar

Agarose is a natural linear polysaccharide derived from the marine red algae *Rhodophyceae*, made from repeating monomeric units of agarobiose (Zarrintaj et al., 2018). In comparison, agar, famously used for microbiological growth purposes, is comprised of a heterogenous mixture of agarobiose and agaropectin. Their features of high biocompatibility, commercial availability, ability to adjust stiffness with concentration and reversible polymerization enabled the use of agar and agarose as *in vitro* and *in vivo* cell scaffolds that mimic soft tissue stiffness (Varoni et al., 2012). Whilst normally inert to cell interaction, agarose can be bioengineered to integrate biofunctional peptides that supports cell adhesion and cell viability (Yamada et al., 2020).

The use of soft agar to determine tumorgenicity and invasiveness of cancer cell lines has been employed for more than 50 years (Shin et al., 1975). Early work utilized soft agar assays where clusters of cancer cells from malignant effusions from ovarian cancer patients were propagated as clones on an agar base and responded to the anti-cancer drugs cisplatin and 5-FU in a similar manner to their derivative patient (Ozols et al., 1980). Agarose, as a hydrogel for ovarian cancer cells, has been used to show physiological cancer characteristics such as elevated expression of HIF-1α, VEGF-A, profibrogenic MMP-2 and -9 when compared to 2D SK-OV-3 monolayers (Xu et al., 2014). In contrast, an agarose coating was used as a non-adherent surface on which the liquid overlay technique of spheroid formation was used. HEY and OVHS1 ovarian cancer cells grown by liquid overlay technique with an agarose low-attachment base, formed spheroids more readily than the normal ovarian cell line IOSE29, and further, showed metastatic features such as disaggregation and MMP activation when transferred onto an ECM (Shield et al., 2007).

#### 5.2.2 Synthetic ECM Hydrogel Scaffolds

Synthetic ECMs are becoming a more popular *in vitro* ECM option compared to gold standard EHS-derived ECMs, due to a higher degree of control over properties such as stiffness, pore size and biofunctionalization.

##### 5.2.2.1 PEG-Based Hydrogels

Hydrogels based on bioinert polyethylene glycol (PEG) have been widely utilized for *in vitro* 3D models for their ability to customize biofunctionalization by peptides and hydrogel stiffness control. The inclusion of MMP-degradable crosslinkers also enables more physiological proteolytic ECM remodeling by cancer cells (Lutolf et al., 2003). A 4-arm PEG-maleimide based hydrogel was designed to recreate the omentum ECM with GFOGER (collagen-I), PHSRN-RGD (fibronectin), RGD (fibronectin, vitronectin, collagen-VI), and DGEA (collagen-IV) biofunctionalization peptide motifs, adjusting stiffness to a physiological level of ∼2.9 kPa for omental tissue and incorporating MMP-degradable crosslinkers (Brooks et al., 2019). This hydrogel supported cell growth and viability of SK-OV-3 multicellular spheroids that were pre-made in non-adherent polydimethylsiloxane (PDMS)-coated grids and spheroids from patient-derived ascites, with paclitaxel and doxorubicin treatments mimicking the drug responses seen in patients. PEG-crosslinked poly(methyl vinyl ether-*alt*-maleic acid) or PEMM/alginate network hydrogels induced EMT, a CSC-like phenotype and chemoresistance in encapsulated SK-OV-3 spheroids, driven by hydrogel stiffness, porosity and cation of choice for crosslinking (Zhou et al., 2020). This pro-metastatic phenotype was confirmed when hydrogels were implanted into immunodeficient mice. An extension of this work by the same authors showed higher RGD (arginine-glycine-aspartic acid) concentrations encouraged spheroid cell dispersion and drug sensitivity, whereas hydrogels with lower RGD concentrations had preserved cell aggregations with CSC-like changes and drug resistance when grown on PEMM hydrogel discs with varying RGD concentrations (Zhou et al., 2021). When taken together to account for mechanical properties, lower stiffness hydrogels with low RGD levels promoted a CSC-like phenotype with drug resistance. While this study highlighted the importance of cell-ECM interactions in drug resistance, the hydrogels studied were of a stiffness much higher than *in vivo* physiological conditions ranging from 60–240 kPa.

##### 5.2.2.2 Gelatin Methacryloyl

GelMA is a semi-synthetic bioengineered, biocompatible material with high batch-to-batch consistency, control of mechanophysical properties, innate RGD responsive peptide motifs and ability to be cleaved by MMPs, having been designed as an alternative to Matrigel (Zhu et al., 2019). GelMA requires modification with a photocrosslinker (typically UV or visible light responsive) for efficient polymerization and to prevent degradation. Visible light, as expected, promotes higher cell viability and lower free radical damage compared to UV light when using GelMA (Noshadi et al., 2017). Addition of Laminin-411 and hyaluronic acid to GelMA hydrogels of 3.4 kPa stiffness enabled ovarian cancer spheroid formation, proliferation and chemoresistance to paclitaxel (Kaemmerer et al., 2014). GelMA hydrogel models also showed similar tumorigenic responses when transplanted into NOD/SCID mice. Interestingly, peripheral blood mononuclear cells or PBMCs grown on GelMA hydrogels showed suppressed pro-inflammatory responses to stimulation with lipopolysaccharide (LPS), particularly in the presence of tumor necrosis factor (TNF), whereby cellular TNF mRNA levels were elevated, but soluble TNF was bound to the hydrogel (Donaldson et al., 2018). This study highlighted the need to consider the immunogenic properties of biomaterials to match hydrogel models for their appropriate application, and may have implications on future 3D *in vitro* immunotherapy models. In the 3D bioprinting field, ovarian cancer cell lines have to date only been used to test printability of GelMA with extrusion based bioprinting, prior to testing its biocompatibility with murine oocytes (Wu et al., 2021).

##### 5.2.2.3 Peptide-Based Hydrogels

Self-assembling peptide-based hydrogels are increasingly being used as a biomimetic material for 3D *in vitro* culture for their engineerability, ability to form well-defined nanofibers with natural amino acid constituents and absence of animal-derived contaminants (Yang et al., 2020). RADA16-I peptide-based hydrogels performed as well as Matrigel and Collagen-I based hydrogels in terms of cell viability, adhesion, migration and drug resistance of encapsulated A2780 and SK-OV-3 ovarian cancer cells (Yang and Zhao, 2011). A disadvantage of some peptide-based hydrogels is their low mechanical strength, where hydrogels with encapsulated cells are able to be disrupted easily by mechanical forces such as pipetting (Song et al., 2020). A study by Hedegaard and colleagues utilized peptide amphiphile-based hydrogels with elastin mimetics, fibronectin, keratin, RGDS (arginine-glycine-aspartic acid-serine) motif for cell adhesion and GHK (glycine-histidine-lysine) motif for pro-angiogenic signaling with encapsulated OVCAR-4 cells in monoculture as well as in co-culture with HUVECs and human mesenchymal stem cells (Hedegaard et al., 2020). Tumor spheroids grown in this hydrogel were comparable in morphology, viability and drug response compared to those grown in GelMA and Matrigel. Importantly, this model is one of the first to incorporate peptides to promote angiogenesis, addressing one of the major missing components of 3D *in vitro* cell culture models.

#### 5.2.3 Decellularized Ovary ECM

Decellularized extracellular matrices (dECM) are the natural matrices from an organ that have been void of all native cellular components, preserving the biological and mechanical properties of the original ECM. These ECMs can be used to seed new cells in an organ’s native conformation in the absence of an immune response, as well as can be lyophilized and reconstituted to form hydrogels. To date, there have been no reports of using decellularized ovary ECM for the study of ovarian cancers. However, the use of ovarian dECMs might benefit 3D *in vitro* ovarian models, due to the preservation of ECM proteins and growth factors found in the native ovary. A handful of studies have shown high biocompatibility with ovarian cell types grown in reconstituted dECM hydrogels and scaffolds. A mixture of sodium alginate with decellularized murine ovarian tissue supported *in vitro* follicle survival (Nikniaz et al., 2021). Preliminary studies of hydrogels derived from decellularized porcine ovarian ECM highlighted the effect of ECM stiffness on ovarian follicle development, with stiffer matrices reducing oocyte viability and triggering premature follicle release (Buckenmeyer et al., 2016), though this effect has not been investigated with ovarian cancer cells. There are nevertheless some concerns surrounding the use of dECMs, namely donor batch differences, retention of native genetic material such as DNA, and the harsh decellularization process that may result in a loss of critical downstream biological interactions with cells (Mendibil et al., 2020).

#### 5.2.4 Organotypic Omental Mesothelial Model

Three-dimensional organotypic models of the human mesothelium (Figure 3) have been utilized for the study of early metastasis to the omentum and interactions with the tumor microenvironment (Kenny et al., 2007). Taking a layered approach to reproduce the architecture of the omentum as observed from histological staining of omental biopsies, this model consists of primary human fibroblasts with ECM, rat-tail collagen-I and human fibronectin, as a base, layered with primary human mesothelial cells. Ovarian cancer cells or ascites-derived spheroids can be added to investigate cell attachment and invasion into the omentum. Using this model, Kenny and colleagues identified MMP-2 mediated cleavage of fibronectin and vitronectin produced by mesothelial cells as an early response to omental metastasis (Kenny et al., 2009). Another study identified mir-193b downregulation as a driver of omental metastasis with this mesothelial model (Mitra et al., 2015a). Further, Henry and colleagues described the dynamic roles of the receptor tyrosine kinases ROR1 and ROR2 in different omental cell types that promote cancer cell adhesion in early metastasis (Henry et al., 2017). This model has also been adapted for high throughput drug screening of potential inhibitors of adhesion and invasion. Though the mesothelial models had fewer efficacious drug hits than traditional 2D models, similar drug responses were observed *in vivo* in PDX models (Kenny et al., 2014; Lal-Nag et al., 2017). This organotypic model was also more cost and time-efficient with similar results to *in vivo* models, when testing micellar-based nanoparticle therapies to prevent early metastasis (Lu et al., 2019). To add complexity to the static organotypic mesothelial model, it has been incorporated into a microfluidics device, enabling the study of hydrodynamic forces of ascites fluid flow on spheroid attachment and metastasis.

The use of the organotypic mesothelial model has been reviewed in depth and a standardized protocol published to enable reproducibility between research groups across the world (Peters et al., 2015; Watters et al., 2018). While this co-culture model has been quite successful, as a scalable, 3D pre-clinical model of ovarian cancer metastases, it lacks vasculature, as well as immune cells and cells from adipose tissue.

#### 5.2.5 3D Ovarian Cancer Co-Cultures

In order to best recapitulate the TME of ovarian cancers, the addition of cancer-associated cells types including stromal, immune, mesenchymal, mesothelial, and endothelial cells, to ovarian cancer cells *in vitro* enables the heterotypic cell-to-cell crosstalk that may influence chemoresistance, angiogenesis, and metastasis. Two-dimensional co-cultures, while enabling intercellular contact and cross-talk, cannot mimic the multidimensional cell interactions. Three-dimensional co-cultures, particularly when combined with ECM scaffolds, are *in vitro*, biomimetic models that may better represent the *in vivo* situation and more accurately predict cellular responses in a patient. While not an exhaustive list, recently published 2D and 3D ovarian cancer co-cultures models are summarized in Table 3.

**TABLE 3:** Indirect and direct *in vitro* ovarian cancer co-culture models.

Co-culture type	Co-culture model	Model format	References
Indirect/non-contact	Cancer-stroma	SK-OV-3 + FP-96 fibroblasts (transwell insert)	Medeiros et al. (2019) ^a^
		OVCAR-5 + MRC-5 fibroblasts (bioprinted onto Matrigel)	Xu et al. (2011)
	Cancer-immune (macrophage)	SK-OV-3, HEY, HO8910, A2780 in Matrigel (transwell insert) + Primary macrophages	Yi et al. (2020)
		SK-OV-3 spheres (transwell insert) + THP-1 macrophages	Ning et al. (2018)
		SK-OV-3 + THP-1 macrophages (transwell insert)	Wang et al. (2013) ^a^
	Cancer-endothelial	SK-OV-3, OVCAR-3 + HUVECs on Matrigel (transwell insert)	Yuan et al. (2021) ^a^
		“NICO-1” transwell system—Primary OvCa stem cell spheroids (ascites, ULA plate) + HUEhT-1 endothelial cells on Matrigel	Miyagawa et al. (2020)
	Cancer-MSC	OvCa cell lines + MSC (adipocyte, bone marrow, umbilical cord) conditioned media	Khalil et al. (2019) ^a^
Direct/contact	Cancer-stroma	SK-OV-3 on top of WI38 fibroblasts in Matrigel	Lau et al. (2017)
		HEY or SK-OV-3 + NIH3T3 cells in hanging drop	Weydert et al. (2020)
		A2780 + Human ovarian fibroblast cell line in a microfluidic chip	Flont et al. (2020)
		SK-OV-3 + mesenchymal cells (MUC-9) or fibroblasts (CCD27-Sk) in ULA plates	Tofani et al. (2021)
	Cancer-immune (macrophage)	ID8 cells on top of Matrigel + TAMs from mouse ascites	Long et al. (2018)
		OVCAR-3 + PBMCs in hanging drop	Raghavan et al. (2019)
	Cancer-adipocyte	ID8 cells on top of primary mouse adipocytes in Matrigel	John et al. (2019)
	Cancer-mesothelial	OVCA433 spheroids (created on poly-HEMA coating) on top of immortalized human lung mesothelial cells or MeT-5A mesothelial cells	Iwanicki et al. (2011)
		CAOV-3 or A2780 + Primary mesothelial cell or MeT-5A mesothelial cell spheroids on poly-HEMA coated plates	Shishido et al. (2018)
		OV-MZ-6 + MeT-5A mesothelial cells in PEG hydrogel	Loessner et al. (2019)
	Organotypic omental mesothelial model	OvCa cell line + Primary mesothelial cells + Primary omental fibroblasts	Kenny et al. (2009); Kenny et al. (2007); Mitra et al. (2015a); Henry et al. (2017); Lu et al. (2019); Li et al. (2017); Peters et al. (2015)
	Multicellular models	OVCAR-4 + HUVEC + hMSC in peptide-based hydrogels	Hedegaard et al. (2020)
		Patient explant orbital rotational cultures (epithelial cells, fibroblasts, tumor-infiltrating immune cells)	Abreu et al. (2020)
		Early passage HGSOC organoids (maintained immune cells)	Hill et al. (2018)

a Cancer cells grown in 2D

MSC, Mesenchymal stem cell; HUVEC, Human umbilical vein endothelial cell; CSC, Cancer Stem Cell; OvCa, Ovarian Cancer; TAM, tumor-associated macrophages; PBMC, primary blood mononuclear cell; PEG, polyethlyene glycol.

There are two major co-culture methodologies: indirect and direct contact models. Indirect contact co-cultures, such as by the use of transwells or conditioned media, mimic cellular signaling in the absence of physical cellular contact, and enable simple segregation of cell types for ease of downstream analyses of individual cell population behavior. Direct contact co-cultures enable the study of physical interaction of several cell types and their influence on cell behavior as an “organ-like” model. Two-dimensional culture studies showed ovarian cancer cell lines in contact with normal omental fibroblasts could drive activation to a cancer-associated fibroblast (CAF) phenotype associated with TGF-β1 secretion (Cai et al., 2012).

In 3D multicellular spheroid cultures, addition of fibroblasts, mesenchymal stem cells, or endothelial cells have been shown to enhance spheroid size, chemoresistance and a stem-like phenotype compared to 3D monocultures (Hedegaard et al., 2020; Tofani et al., 2021). Co-cultures of ovarian cancer cells and macrophages have identified positive feedback loops that drive stemness, chemoresistance and spheroid formation in cancer cells and M2 polarization of macrophages (Long et al., 2018; Ning et al., 2018; Raghavan et al., 2019). Three-dimensional co-culture systems of ovarian cancers and mesothelial cells, including the organotypic omental mesothelium model (described in Section 5.2.4) have also enabled the study of mechanisms driving peritoneal and omental metastasis (Iwanicki et al., 2011; Henry et al., 2017; Shishido et al., 2018; Loessner et al., 2019).

The number of studies utilizing 3D co-cultures of ovarian cancers at the time of this publication was limited but is growing. It is clear that with increasing model complexity through addition of multiple patient-derived cell types in conjunction with physiologically relevant ECM mimics, that the most biologically appropriate and predictive *in vitro* models will be discovered. Integration of organoids with endothelial cells could enable more biologically appropriate, predictive studies of anti-angiogenic drugs such as bevicizumab in the presence of ovarian cancers with specific mutations. Further, utilization of microfluidics devices for emulation of fluid flow and the addition of immune cells types enable the creation of a more accurate biomimetic TME.

#### 5.2.6 Organoids

Patient-derived organoids are becoming an important and powerful pre-clinical model for personalized medicine (Figure 3). While primary patient-derived samples are the gold standard for prediction of treatment response, long-term culture of these samples in 2D often leads to phenotypic changes and differential responses to drug treatments (Kapałczyńska et al., 2018). Cryopreservation of patient-derived organoids allows for biobanking of unique samples for genotype/phenotype matching with future samples from the same patient as their cancer progresses to inform treatment decision making.

In comparison to xenograft models, organoid or tumoroid cultures require significantly shorter times for development from small starting samples, have a higher success rate and accurately reproduce the genetic and phenotypic features of the derivative tumor, allowing for personalized medicine strategies (Hidalgo et al., 2014). Nanki and colleagues were able to propagate primary ovarian tumor organoids from a variety of histological subtypes including HGSOC, OCCC, and EnOC with an 80% success rate and maintenance of their original tumor mutational profiles (Nanki et al., 2020). The analysis of ovarian tumor organoids derived from multiple tumor sites within the same patient showed differential drug sensitivities, emphasizing the need to account for intrapatient tumor heterogeneity (De Witte et al., 2020). When grown in a modified Matrigel bilayer, organoids from MOCs could reproduce behaviors of their derivative cancer, such as production of mucin and a cystic morphology (Maru et al., 2019).

While there is growing evidence that ovarian cancer organoids/tumoroids are becoming the new benchmark for pre-clinical models compared to traditional 2D methods, varying success rates have been reported, particularly attributed to the heterogeneity of these cancers, and propagation success has been highly dependent on starting sample volume and sample handling. In contrast to traditional organoid culture methods that utilize Wnt to maintain stem-like properties, Wnt pathway induction resulted in tumoroid growth arrest in HGSOC organoids, however was able to be rescued by BMP signaling (Hoffmann et al., 2020). The opposite was observed in fallopian tube organoids whereby shRNA downregulation of tumor suppressor genes *TP53*, *PTEN,* and *RB*, showed stem-like changes occurring early in tumor development. Work by Maenhoudt and colleagues identified that the addition of neuregulin-1 (NRG1) to culture medium can increase the proliferation time of primary organoid cultures from fresh and cryopreserved HGSOC and LGSOC, particularly in slower-growing cultures (Maenhoudt et al., 2020). In contrast, it has been noted that organoid propagation methods tend to be selective and result in a cell population that may not be representative of the original tumor heterogeneity and in turn, treatment response, particularly in patients who have undergone previous neoadjuvant therapy (Hill et al., 2018). Therefore, care must be taken in interpretation of data and use of the correct modelling systems. Improvements in methodologies to maintain the heterogenous phenotype that exists in patient tumors are needed, such as propagation of organoids from different regions of the same tumor (Maru et al., 2019). Furthermore, the standardization of protocols and materials for organoid culture between laboratories around the world will lead to vast improvements in reproducibility and reliability of this technique into the future.

As the initial triggers for the development of ovarian cancer is still being debated, organoids from normal ovarian tissue have also been utilized in studies of tumor development. Organoid cultures from high-risk women with germline mutations in *BRCA1* or *BRCA2* have been developed for research into early tumor development (Kopper et al., 2019). Kopper and colleagues have performed gene editing in normal non-tumor organoids established from fallopian tube or ovarian epithelium using CRISPR-Cas9 gene editing for modelling *TP53* mutations, and determined that HRD ovarian cancer organoids with fewer RAD51 loci were more sensitive to the PARPi niraparib, analogous to responses observed *in vivo* (Kopper et al., 2019). An extension of this work has similarly used CRISPR-Cas9 genome editing to introduce common HGSOC gene defects into mouse oviduct and OSE cell organoids, showing that both organoid types could become carcinogenic and that drug sensitivities in HGSOC patients may be cell lineage-dependent (Lõhmussaar et al., 2020).

### 5.3 Bioprinting of 3D OC Models

Three dimensional bioprinting presents an automated solution that combines both ECM components and high-throughput creation of ovarian cancer models in a spatially-controlled manner (Figure 3). As a relatively new approach to 3D cell model creation, the number of published studies is limited for ovarian cancer.

Droplet-based bioprinting systems enable the precise and agile fabrication of microtissue cultures by overlaying drops of biomaterial. Used mainly for deposition of scaffolds, this layer-by-layer technique has also been used for addition of drugs, growth factors and living cells. Xu and colleagues have used a droplet-based system to bioprint OVCAR-5 cells and MRF-5 normal fibroblasts on top of a Matrigel scaffold in a controlled spatial distribution for investigation of feedback mechanisms between tumor and stromal cells (Xu et al., 2011). While there are no publications to date utilizing drop-on-demand inkjet bioprinting, laser-assisted bioprinting or stereolithography with ovarian cancer cells, these are promising techniques that could enable high-throughput 3D ovarian cancer cell model development that would seamlessly integrate with existing high-throughput drug screening technologies (Mazzocchi et al., 2019).

Extrusion based bioprinting has been employed to fabricate a 3D bioprinted ovary that successfully supported oocyte growth using a GelMA-based bioink (Wu et al., 2021). While this study focused on oocyte maturation *ex vivo*, ovarian cancer cell lines were utilized during optimization of bioink biocompatibility, showing high cell viability during and after the extrusion process. Unfortunately, this was not translatable in primary murine oocytes, suggestive of the delicate nature of primary-derived cells with this technique.

An important factor of 3D bioprinting is the printability of the matrix to be deposited. The material properties of Matrigel and other natural matrices are limited in their printability as bioinks due to temperature sensitivities and pre-determined stiffnesses, highlighting the flexibility of synthetic matrices that can be modified to best mimic *in vivo* conditions. In contrast, synthetic matrices are limited by their biocompatibility but can be biofunctionalized with peptides and full-length ECM proteins. Overall, 3D bioprinting and automation has great potential as a future staple for *in vitro* and *ex vivo* studies driving drug development and discovery for all cancer types.

### 5.4 Bioreactors

Bioreactors have largely been used to study the effects of fluid flow as a physiological mechanical stimulus on cell behavior. In the context of ovarian cancers, mechanotransduction and shear stress from ascites fluid build-up and flow in the peritoneum have been shown to drive ovarian cancer metastasis. Tumor-on-a-chip microfluidics systems have been used to model peritoneal metastases as well as vasculature in solid tumors.

#### 5.4.1 Rotating Wall Vessels and Orbital Shakers

Rotating wall vessel bioreactors or rotary cell culture systems (Figure 2) utilize low shear, low turbulence biomechanical forces to influence cell differentiation and aggregation in three dimensions as a suspension (Schwarz et al., 1992). Originally designed to mimic microgravity, this technique has been important for studies of transcoelomic ovarian cancer metastases (Shield et al., 2009). Microgravity, as a biomechanical force, has been reported to reduce metastatic markers, change cell metabolism and chemosensitivity in a variety of cancer cell lines, though the *in vivo* effect is unknown (Takeda et al., 2009; Vidyasekar et al., 2015). LN1 cells, derived from a mixed Mullerian tumor, were grown in a horizontally-oriented high aspect rotating vessel (HARV) with microcarrier beads as a scaffold to investigate the potential for metastatic growth as spheroids or aggregates (Becker et al., 1993). This study also showed that there is growth selection for certain cell types from a mixed population in 3D compared to 2D. Further work confirmed the production of chondroitin sulphate in the 3D culture similar to that observed in the *in vivo* tumor; however, this was not observed in 2D culture (Goodwin et al., 1997; Grun et al., 2009). These spheroids also showed varying degree of oncogene expression not seen in 2D, although the variation was likely due to the mixed populations present. Low passage primary ovarian and endometrial cancer cell lines propagated as multicellular spheroids in a rotating cell culture system, showed similar histological markers to the primary tumor with differentially expressed markers including prohibitin, VDAC1 and annexin 4 identified in 2D *versus* 3D culture methods (Grun et al., 2009).

Orbital shakers, traditionally used for bacterial cultures, have also been employed to investigate fluid shear stress on ovarian cancer spheroid formation. Masiello and colleagues adapted the orbital shaker to rotate at physiologically relevant speeds and utilized typical culture dishes to investigate the effects of rotational speeds, cell density and well size on spheroid formation (Masiello et al., 2018). This study resulted in more rounded and consistent spheroid formation, amenable to the analyses of functional endpoints. Patient-derived explants of multiple ovarian cancer histotypes have been propagated for up to 30 days in orbital shakers, resulting in maintenance of tumor architectures, epithelial, fibroblasts and immune cells, as well as ECM (Abreu et al., 2020). Studies using these rotational techniques have contributed to the understanding of shear stress on ovarian cancer metastasis, drivers of spheroid formation in ascites and long-term propagation of ovarian cancer cells.

#### 5.4.2 Compressive Stress Bioreactors

The majority of bioreactor-based models for ovarian cancer have been used for the study of fluid shear stress pressures on cancer invasion and proliferation. Investigation into compressive stress to model external compressive stimuli such as ascites accumulation that increases hydrostatic pressure as a driver of metastasis is a relatively new approach and its effect on treatment is still unknown. Novak and colleagues created a custom bioreactor to mimic the hydrostatic compression pressures on ovarian cancer metastasis from the primary tumor (Novak et al., 2020). This was modelled by the HGSOC cell line OVSAHO that was encapsulated in an agarose-collagen-I hydrogel on top of a membrane where air can be pumped to mimic the compressive forces from ascites fluid build-up in both a static and cyclic manner. This work identified CDC42 as a driver of chemoresistance and proliferation under compressive stimuli. Recently, Onal and colleagues developed a microfluidic platform with micro-pistons that enable the application of dynamic compression to the system, for investigations of cyclic and pressure-controlled compressive stress on SK-OV-3 cell damage (Onal et al., 2021). While this study used ovarian cancer cells as a monolayer, the authors iterate that their platform can be easily modified to include hydrogel ECM and spheroid models.

#### 5.4.3 Tumor-on-a-Chip Microfluidic Systems

Tumor-on-a-chip devices utilize microfluidics to simulate effects of vascular flow in solid tumors and more specifically in the context of ovarian cancer, mimicking the hydrodynamics that influence ascitic seeding in the peritoneum and contributing to metastases (Figure 3). Microfluidic devices consist of networks of microchannels where fluids including cell media with cells, or media with drugs or cytokines can be injected and evacuated in an automated manner, enabling nutrient and gas exchange. Microchannels may be laden with matrix and/or cells to mimic a lumen. Culture within a microfluidic system is often short-term and high-throughput.

Tumor-on-a-chip microfluidic systems have been used to model various aspects of ovarian cancer progression, both as solid primary tumors and drivers of cell survival in metastases. Microfluidic chips have been used to model 3D tumor nodule development in the peritoneum by ascitic spheroids, showing that the flow stream was a factor that drove OVCAR-5 cell attachment to Matrigel-laden walls *via* EMT (Rizvi et al., 2013). Using the same system, SK-OV-3 spheroids were flowed through a poly-HEMA-coated chamber to investigate the effects of shear stress on EMT status changes in cancer spheroids, similarly showing that perfusion promoted spheroid viability and stemness (Ip et al., 2016). These findings were further validated when transplanted into nude mice as xenografts, with perfused spheroids forming larger subcutaneous tumors that were found to be chemoresistant, regulated by the PI3K/AKT signaling pathway. Microfluidic chips have also been used to investigate platelet extravasion in the presence of both primary endothelial cell and ovarian cancer cells, whereby platelets were found to induce cancer cell mediated disruption of the endothelial layer (Saha et al., 2020). This disruption was partially rescued by addition of atorvastatin, a statin drug used for preservation of endothelial junctions, with results reproduced using primary HGSOC cells with prior atorvastatin treatment *in vivo*. Induction of inflammatory cytokines such as MCP-1 and TNF also mirrored the pro-inflammatory vascular microenvironment observed in the vicinity of ovarian tumors *in vivo* and inferred some regulation of cancer progression.

Advantages of microfluidic chip systems include the ability to utilize small amounts of starting material, homogenous creation of large numbers of spheroids, portable sizing, and the potential to control drug or chemokine gradients with integration of multiple wells or chips. An orthogonal microfluidic chip developed for seeding into PDMS-based microwells, was superior to standard low-attachment plates and Matrigel seeding in cell viability and maintenance of patient-derived epithelial ovarian cancer cell phenotypes, even when starting with low starting cell numbers (Dadgar et al., 2020). The multi-well microfluidic chip also enabled simpler cytotoxicity measurements by imaging analyses when compared to Matrigel methods, and allowed simultaneous drug gradients in a single microfluidic chip. A direct comparison of spheroid formation and carboplatin sensitivity in four ovarian cancer cell lines in either PDMS-based microfluidic devices with microwells, ULA plates or hanging drop showed microfluidic devices created more homogeneous spheroids with similar carboplatin sensitivities to ULA-plate derived spheroids (Patra et al., 2020). Microfluidic systems have also been used to study macrophage recruitment by ovarian cancer cells *via* integration of chemokine concentration gradients to mimic macrophage infiltration into the tumor with both HGSOC cell lines and patient-derived xenografts (Scott et al., 2021). The study showed correlation between macrophage recruitment and tumor invasiveness and results were replicated in *in vivo* PDX models, with future work looking to identify correlations between macrophage infiltration and chemoresistance.

While microfluidic devices have great potential as more physiological and reproducible 3D *in vitro* models of ovarian cancer, there are still a number of drawbacks to this new technology, namely in costing. There is also a high level of variability between studies due to the majority of devices being fabricated in-house to meet specific researchers’ needs, though commercially available options ranging from single to multichannel perfusion systems are becoming increasingly obtainable, that assists with method standardization.

## 6 3D *in vitro* Ovarian Cancer Models for Personalized Medicine

The concept of a personalized medicine approach where specific treatments are tailored for individuals, is the overarching goal of cancer therapy. As already discussed, ovarian cancer is in fact an umbrella term for multiple histotypes, with differing sites of origin, different genetic and epigenetic events and survival rates. Further, ethnicity and different occurrence rates of histotypes between ethnicities adds another layer of complexity (Peres et al., 2018). Given these variables, homogenous, immortalized 2D cell lines as *in vitro* models of ovarian cancer only address few of these variables at a single given timepoint of the disease and in a defined microenvironment dissimilar to the *in vivo* situation. Therefore, there is a need for more representative *in vitro* and *ex vivo* models of ovarian cancer for a more personalized prediction of therapeutic efficacy.

There is growing evidence that 2D cultures are more divergent and 3D cultures are better models to reproduce and predict patient drug responses. Patient-derived spheroids grown in 384-well ULA plates derived from primary debulking surgery formed over 24 h and after a further 72 h of growth predicted patient response to first-line adjuvant chemotherapies with 89% accuracy (Shuford et al., 2019). A study by Jabs and colleagues documented that primary ovarian cancers cells grown as organoids had HRD scores, growth and drug cytotoxicity more strongly correlated with the original tumor compared to monolayer cultures, indicative that 3D tumoroid cultures are better mimics of patient tumor behavior (Jabs et al., 2017). Further, numerous studies have shown the utility of ovarian cancer organoids of various histotypes for drug screening, with maintenance of mutational profiles and accurate reproduction of treatment responses when challenged with FDA-approved clinically utilized drugs (Kopper et al., 2019; Maenhoudt et al., 2020; Nanki et al., 2020).

For the prediction of drug response, HGSOC tumor organoids from primary and interval debulking surgeries showed histological concordance with the original tumor and reliably predicted carboplatin sensitivity and resistance (Gorski et al., 2021). RNA sequencing of these tumoroids identified cell-specific pathways that may contribute to carboplatin sensitivity or resistance, and has potential for use in stratification of patients, to guide treatment strategies before clinical recurrence (Gorski et al., 2021). High-throughput development of patient-derived ovarian cancer organoids grown in a ring-shaped geometry of Matrigel, was able to identify responses to 240 kinase inhibitor compounds within a week of cell isolation (Phan et al., 2019). The ring-shaped geometry is particularly advantageous as it eliminates the need for sample transfer or tumoroid dissociation, and can utilize very small starting cell numbers. Further, tumoroids grown by this method were able to maintain heterogeneity with distinct cytomorphologies from mixed type carcinomas. This method could enable rapid screening and identification of clinically actionable drugs as well as identify clinical trial eligibility. Hill and colleagues employed organoids from HGSOC for DNA repair profiling and were able to predict the therapeutic sensitivity in patients to PARP inhibitors. This valuable study highlights the use of organoids as a tool to guide treatment decisions that may have the most benefit (Hill et al., 2018).

## 7 Future Perspectives

**FIGURE 4:**
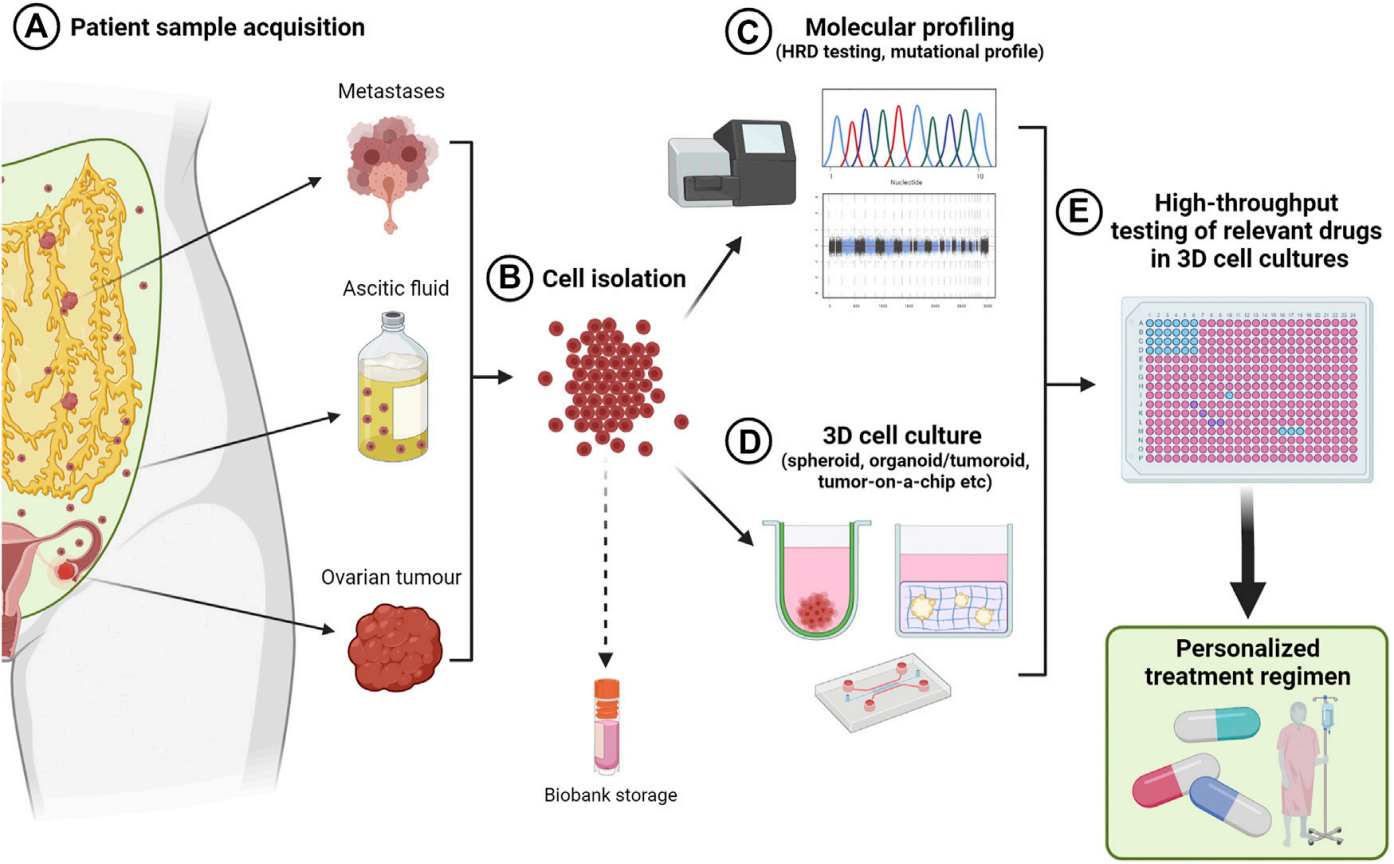
A bench-to-bedside approach using 3D cell cultures to fast track personalized therapies for ovarian cancers. Utilization of **(A)** samples from multiple patient tumor sites, **(B)** isolation of cancer cells *ex vivo* for **(C)** molecular profiling and **(D)** propagation as 3D cell cultures can identify clues regarding a patient’s unique tumor phenotype. Based on these findings, **(E)** a high-throughput drug screen of molecularly relevant drugs in 3D cell cultures can be employed to predict drug efficacy and utilized to guide a personalized medicine approach. Created with Biorender.com.

Given the high mortality and recurrence rate of ovarian cancers, there is a clear need for more physiologically relevant models that can accurately predict the likelihood that a patient will respond to a particular therapy, both at diagnosis and relapse in order to guide therapeutic strategy. These models will need to be constructed across all of the ovarian cancer histotypes, including those that are very rare, and generate results in a timeframe that facilitates rapid and targeted translation to patients. A vision for the future would encompass automated functional assays of ovarian tumoroids including tumor proliferation, metastatic spread, spheroid formation and cell death that routinely complement molecular studies to provide strong predictions of patient response to new molecular targeted therapies such as described in Figure 4. Furthermore, discovery-based science that will identify new drugs to treat ovarian cancer, will also be conducted in models that more strongly mimic *in vivo* condition. These studies will not only identify new therapies, but also diagnostic and prognostic markers.

The field of 3D bioprinting, currently in its infancy, is rapidly emerging as an answer to the manual, low throughput methods of creating 3D cell models, including organoids and tumoroids. In the future, the challenges of including common components missing in most 3D *in vitro* models today of ovarian cancer such as incorporation of vasculature, immune cells and hydrostatic forces will be met as models are created to best recapitulate the patient environment and to better inform the clinical management of women with ovarian cancer.
