# Supplementary material for: Three-Dimensional Modelling of Ovarian Cancer: From Cell Lines to Organoids for Discovery and Personalized Medicine
Source: Front Bioeng Biotechnol. 2022 Feb 10;10:836984. doi: 10.3389/fbioe.2022.836984 (PMC8866972; doi:10.3389/fbioe.2022.836984)
Supplement: Supplementary file 1 [file DataSheet1.docx]

**Supplementary Table 1.** **Ovarian cancer cell line origin, *in vivo* growth and classifications.**

| **Cell line** | **OvCa Histotype** | **Specimen site** | **Growth *in vivo* in mice** | **Commercial availability** | **References** |
| --- | --- | --- | --- | --- | --- |
| FU-OV-1 | HGSOC ^a, d, e^ | Ovary tumor tissue | Unknown | DSMZ | Emoto et al. (1999)^ |
| JHOS-2 | HGSOC ^a, d, e^ | Ovary tumor tissue | Unknown | RIKEN BRC | Yamada et al. (1999)^ |
| JHOS-4 | HGSOC ^a, d, e^ | Metastatic site: Peritoneum | Unknown | RIKEN BRC | Unpublished data, originated by Ishikawa H and Yasuda M. |
| OV167 | HGSOC ^e^ | Primary tumor | Unknown | No | Conover et al. (1998)^ |
| PEO6 | HGSOC ^e^ | Ascites | Unknown | ECACC | Langdon et al. (1988)^ |
| SNU-119 | HGSOC ^a, d^ | Ascites | No: SC or IP | KCLB | Yuan et al. (1997)^, Mitra et al. (2015)# |
| SNU-8 | HGSOC ^a, d^ | Ascites | Unknown | KCLB | Yuan et al. (1997)^ |
| EFO-27 * | EnOC ^a, d^ | Omentum; solid omental metastasis | Unknown | DSMZ | Simon et al. (1983)^; Kunzmann and Hölzel (1987)^ |
| OVK18 | EnOC ^a, d, e^ | Ascites | Yes: SC and IP, forms ascites | RIKEN BRC | Uehara et al. (1984)^; De Haven Brandon et al. (2020)# |
| JHOC-5 | OCCC ^a, b, d^ | Pelvis | Yes: SC and IP, forms ascites | RIKEN BRC | Yamada et al. (1999)^; De Haven Brandon et al. (2020)# |
| OV207 | OCCC ^e^ | Primary tumor | Unknown | No | Conover et al. (1998)^ |
| OVHS1 | OCCC | Unspecified | Yes: SC | No | Ohta et al. (1998)^; Shafren et al. (2005) |
| RMG-II | OCCC ^b^ | Ascites | Yes: SC | JCRB | Nozawa et al. (1991)^; Sasano et al. (2015)# |
| VOA1312_CL | LGSOC | Ascites | Unknown | No | Anglesio et al. (2013)^ |
| VOA1056_CL | LGSOC | Primary tumor | Unknown | No | Anglesio et al. (2013)^ |
| CAISMOV24 | LGSOC | Ascites | Unknown | No | da Silva et al. (2017)^ |
| COV644 | MOC ^c, d^ | Primary tumor | Unknown | ECACC | van den Berg-Bakker et al. (1993)^ |
| HTOG | GCT | Granulosa-theca cell tumor | Unknown | No | Ishiwata et al. (1984)^ |
| BIN67 | SCCOHT | Solid primary tumor | Yes: IP | No | Gamwell et al. (2013)^, # |
| SCCOHT1 | SCCOHT | Solid primary tumor | Yes: SC | No | Otte et al. (2012)^, # |

Note: Cell lines identified with <50 publications *via* PUBMED on 10/12/2021.

OvCa (Ovarian Cancer), JCRB (Japanese Cancer Research Resources Bank), KCLB (Korean Cell Line Bank), ECACC (European Collection of Authenticated Cell Cultures), RIKEN BRC (RIKEN BioResource Center Cell Bank), DSMZ (Leibniz Institute, German Collection of Microorganisms and Cell Cultures)

SC (subcutaneous), IP (intraperitoneal), IB (intrabursal) * originally classified HGSOC

Recent classification of histotypes: a. Domcke et al. (2013), b. Anglesio et al. (2013), c. Beauford et al. (2015), d. Barnes et al. (2021), e. Papp et al. (2018)

^ original subtype reference, # *in vivo* tumor growth in mice reference

EnOC (Endometrioid Ovarian Cancer), OCCC (Ovarian Clear Cell Carcinoma), LGSOC (Low Grade Serous Ovarian Cancer), MOC (Mucinous Ovarian Cancer, HGSOC (High Grade Serous Ovarian Cancer), GCT (Granulosa Cell Tumor), SCCOHT (Small Cell Carcinoma of the Ovary, Hypercalcemic Type)

**Supplementary Table 2.** **Ovarian cancer cell lines with multiple histotype classifications.**

| **Cell line** | **OvCa Histotype** | **Specimen site** | **Growth *in vivo* in mice** | **Commercial availability** | **References** |
| --- | --- | --- | --- | --- | --- |
| 59M | EnOC with clear cell component ^c^ (HGSOC ^a^; LGSOC ^d^) | Ascites | Unknown | ECACC | Wilson et al. (1996)^ |
| EFO-21 | HGSOC (possibly HGSOC ^a^; OCCC ^c, d^) | Ascites | Yes: SC | DSMZ | Simon et al. (1983)^; Huang et al. (2013)# |
| ES-2 | OCCC ^e^ (EnOC ^b,c^; LGSOC ^d^) | Tumor tissue | Yes: SC | ATCC | Lau et al. (1989)^; Kashiyama et al. (2014) # |
| HEY A8 | HGSOC ^e^ (unlikely HGSOC ^a,b^; LGSOC ^d^) | Peritoneal deposit; ascites | Yes: SC and IP, forms ascites | ATCC | Buick et al. (1985)^; Hernandez et al. (2016)# |
| IGROV1 | Mixed: EnOC, HGSOC, OCCC, undifferentiated components (hyper mutated ^a^; OCCC ^d^) | Tumor tissue | Yes: SC, IP | MERCK Millipore | Bénard et al. (1985)^; Hernandez et al. (2016)#; De Haven Brandon et al. (2020)# |
| JHOM-1 | MOC ^e^ (HGSOC ^a^; LGSOC ^d^) | NS | Unknown | RIKEN BRC | Unpublished data, originated by Ishikawa H and Yasuda M. |
| JHOM-2B | MOC ^d^ (HGSOC ^a^) | NS | Unknown | RIKEN BRC | Unpublished data, originated by Ishikawa H and Yasuda M. |
| OAW42 | HGSOC ^e^ (unlikely HGSOC ^a^; OCCC ^d^) | Ascites | Unknown | ECACC | Wilson et al. (1996)^ |
| OV56 | HGSOC (unlikely HGSOC ^a^; OCCC/EnOC ^c^; LGSOC/OCCC ^d^) | Ascites | Unknown | ECACC | Boocock et al. (1995)^ |
| OV-90 | HGSOC ^e^ (MOC ^d^) | Ascites | Yes: IP, IB, No: SC | ATCC | Lounis et al. (1998)^; Hernandez et al. (2016)# |
| OVCAR-5* | Unclassified ^e^ | Ascites | Yes: SC and IP | MERCK Millipore | Hamilton et al. (1984)^; Blayney et al. (2016)^; Hernandez et al. (2016)# |
| OVCAR-8 | HGSOC (possibly HGSOC ^a^; HGSOC/EnOC ^b^; LGSOC ^d^) | NS | Yes: SC, IP and IB | No | Schilder et al. (1990); Hernandez et al. (2016)# |
| SK-OV-3 | HGSOC ^b^ (unlikely HGSOC ^a^; OCCC/EnOC ^c^; OCCC ^d^) | Ascites | Yes: SC and IP, forms ascites | ATCC; ECACC | Buick et al. (1985) ^; Hernandez et al. (2016)#, De Haven Brandon et al. (2020)# |
| TYK-nu | HGSOC ^a^ (LGSOC ^d^) | Ovary | Unknown | JCRB | Yoshiya (1986)^ |

Note: OvCa (Ovarian Cancer), Not specified (NS), ATCC (American Type Culture Collection), JCRB (Japanese Cancer Research Resources Bank), ECACC (European Collection of Authenticated Cell Cultures), RIKEN BRC (RIKEN BioResource Center Cell Bank), DSMZ (Leibniz Institute, German Collection of Microorganisms and Cell Cultures)

SC (subcutaneous), IP (intraperitoneal), IB (intrabursal), * originally classified HGSOC

Recent classification of histotypes: a. Domcke et al. (2013), b. Anglesio et al. (2013), c. Beauford et al. (2015), d. Barnes et al. (2021), e. Papp et al. (2018)

^ original subtype reference, # *in vivo* tumor growth in mice reference

EnOC (Endometrioid Ovarian Cancer), OCCC (Ovarian Clear Cell Carcinoma), LGSOC (Low Grade Serous Ovarian Cancer), MOC (Mucinous Ovarian Cancer, HGSOC (High Grade Serous Ovarian Cancer).

**Supplementary Table 3.** **Immortalized cell lines of normal fallopian tube and ovarian origin.**

| **Cell line** | **Cell type** | **Transformation method** | **References** |
| --- | --- | --- | --- |
| FT33-TAg cells | FTSEC | Fallopian tube transformed with Ras or MYC | Karst et al. (2011) |
| OSE(tsT) | OSE | Ovary transformed with temperature sensitive SV40 large T antigen | Kalli et al. (2004) |
| 1431-E6/E7 | OSE | Ovary transformed by overexpressing HPV-E6/E7 | Shin et al. (2018) |
| 1431-SV40 | OSE | Ovary transformed with SV40 T antigen | Shin et al. (2018) |
| 0160 SV40 | OSE | Ovary transformed with SV40 T antigen | Shin et al. (2018) |
| 8695 SV40 | OSE | Ovary transformed with SV40 T antigen | Shin et al. (2018) |
| 4138 SV40 | OSE | Ovary transformed with SV40 T antigen | Shin et al. (2018) |
| HGrC1 | NLGC | Transformed with Human papillomavirus type 16 (HPV16) protein 6/E7, cyclinD1, mutant CDK4 and hTERT | Bayasula et al. (2012) |
| HO-23 | LGC | Luteinized granulosa cells (LGC) transformed SV40 DNA, Ha-ras oncogene, and a temperature sensitive (ts) mutant of the tumor suppressor gene p53 (p53val135) | Hosokawa et al. (1998) |
| HGL5 | LGC | Luteinized granulosa cells (LGC) transformed with SV40 | Rainey et al. (1994) |
| GC1a | GC | Developing follicles transformed with SV40 | Okamura et al. (2003) |
| HGP53 | GC | Pre-ovulatory human granulosa cells transformed with mutated p53 (p53val135) and Ha-ras genes | Tajima et al. (2002) |

Note: FTSEC (fallopian tube secretory epithelial cells), OSE (ovarian surface epithelium), GC (granulosa cells), NLGC (non-luteinized granulosa cells), LGC (luteinized granulosa cells), SV40 (simian virus 40)

**References**

Anglesio, M. S., Wiegand, K. C., Melnyk, N., Chow, C., Salamanca, C., Prentice, L. M., Senz, J., Yang, W., Spillman, M. A., Cochrane, D. R., Shumansky, K., Shah, S. P., Kalloger, S. E., & Huntsman, D. G. (2013). Type-specific cell line models for type-specific ovarian cancer research. *PLoS ONE*, *8*(9), e72162-e72162. <https://doi.org/10.1371/journal.pone.0072162>

Bayasula, Iwase, A., Kiyono, T., Takikawa, S., Goto, M., Nakamura, T., Nagatomo, Y., Nakahara, T., Kotani, T., Kobayashi, H., Kondo, M., Manabe, S., & Kikkawa, F. (2012). Establishment of a Human Nonluteinized Granulosa Cell Line that Transitions from the Gonadotropin-Independent to the Gonadotropin-Dependent Status. *Endocrinology*, *153*(6), 2851-2860. <https://doi.org/10.1210/en.2011-1810>

Blayney, J. K., Davison, T., McCabe, N., Walker, S., Keating, K., Delaney, T., Greenan, C., Williams, A. R., McCluggage, W. G., Capes-Davis, A., Harkin, D. P., Gourley, C., & Kennedy, R. D. (2016). Prior knowledge transfer across transcriptional data sets and technologies using compositional statistics yields new mislabelled ovarian cell line. *Nucleic Acids Res*, *44*(17), e137. <https://doi.org/10.1093/nar/gkw578>

Boocock, C. A., Charnock-Jones, D. S., Sharkey, A. M., McLaren, J., Barker, P. J., Wright, K. A., Twentyman, P. R., & Smith, S. K. (1995). Expression of vascular endothelial growth factor and its receptors flt and KDR in ovarian carcinoma. *J Natl Cancer Inst*, *87*(7), 506-516. <https://doi.org/10.1093/jnci/87.7.506>

Buick, R. N., Pullano, R., & Trent, J. M. (1985). Comparative properties of five human ovarian adenocarcinoma cell lines. *Cancer Res*, *45*(8), 3668-3676.

Conover, C. A., Hartmann, L. C., Bradley, S., Stalboerger, P., Klee, G. G., Kalli, K. R., & Jenkins, R. B. (1998). Biological characterization of human epithelial ovarian carcinoma cells in primary culture: the insulin-like growth factor system. *Exp Cell Res*, *238*(2), 439-449. <https://doi.org/10.1006/excr.1997.3861>

da Silva, R. F., Yoshida, A., Cardozo, D. M., Jales, R. M., Paust, S., Derchain, S., & Guimarães, F. (2017). Natural Killer Cells Response to IL-2 Stimulation Is Distinct between Ascites with the Presence or Absence of Malignant Cells in Ovarian Cancer Patients. *Int J Mol Sci*, *18*(5). <https://doi.org/10.3390/ijms18050856>

De Haven Brandon, A., Box, G., Hallsworth, A., Court, W., Matthews, N., Herodek, B., Arteagabeitia, A. B., Valenti, M., & Kirkin, V. (2020). Identification of ovarian high-grade serous carcinoma cell lines that show estrogen-sensitive growth as xenografts in immunocompromised mice. *Scientific Reports*, *10*(1). <https://doi.org/10.1038/s41598-020-67533-1>

Emoto, M., Oshima, K., Ishiguro, M., Iwasaki, H., Kawarabayashi, T., & Kikuchi, M. (1999). Establishment and characterization of a serous papillary adenocarcinoma cell line of the human ovary in a serum-free culture. *Pathol Res Pract*, *195*(4), 237-242.

Gamwell, L. F., Gambaro, K., Merziotis, M., Crane, C., Arcand, S. L., Bourada, V., Davis, C., Squire, J. A., Huntsman, D. G., Tonin, P. N., & Vanderhyden, B. C. (2013). Small cell ovarian carcinoma: genomic stability and responsiveness to therapeutics. *Orphanet journal of rare diseases*, *8*, 33-33. <https://doi.org/10.1186/1750-1172-8-33>

Hamilton, T. C., Young, R. C., & Ozols, R. F. (1984). Experimental model systems of ovarian cancer: applications to the design and evaluation of new treatment approaches. *Semin Oncol*, *11*(3), 285-298.

Hernandez, L., Kim, M. K., Lyle, L. T., Bunch, K. P., House, C. D., Ning, F., Noonan, A. M., & Annunziata, C. M. (2016). Characterization of ovarian cancer cell lines as in vivo models for preclinical studies. *Gynecologic Oncology*, *142*(2), 332-340. <https://doi.org/10.1016/j.ygyno.2016.05.028>

Hosokawa, K., Dantes, A., Schere-Levy, C., Barash, A., Yoshida, Y., Kotsuji, F., Vlodavsky, I., & Amsterdam, A. (1998). Induction of Ad4BP/SF-1, steroidogenic acute regulatory protein, and cytochrome P450scc enzyme system expression in newly established human granulosa cell lines. *Endocrinology*, *139*(11), 4679-4687. <https://doi.org/10.1210/endo.139.11.6279>

Huang, L., Wang, H. Y., Li, J. D., Wang, J. H., Zhou, Y., Luo, R. Z., Yun, J. P., Zhang, Y., Jia, W. H., & Zheng, M. (2013). KPNA2 promotes cell proliferation and tumorigenicity in epithelial ovarian carcinoma through upregulation of c-Myc and downregulation of FOXO3a. *Cell death & disease*, *4*(8), e745-e745. <https://doi.org/10.1038/cddis.2013.256>

Ishiwata, I., Ishiwata, C., Soma, M., Kobayashi, N., & Ishikawa, H. (1984). Establishment and characterization of an estrogen-producing human ovarian granulosa tumor cell line. *J Natl Cancer Inst*, *72*(4), 789-800.

Kalli, K. R., Chen, B. K., Bale, L. K., Gernand, E., Overgaard, M. T., Oxvig, C., Cliby, W. A., & Conover, C. A. (2004). Pregnancy-associated plasma protein-A (PAPP-A) expression and insulin-like growth factor binding protein-4 protease activity in normal and malignant ovarian surface epithelial cells. *Int J Cancer*, *110*(5), 633-640. <https://doi.org/10.1002/ijc.20185>

Karst, A. M., Levanon, K., & Drapkin, R. (2011). Modeling high-grade serous ovarian carcinogenesis from the fallopian tube. *Proceedings of the National Academy of Sciences*, *108*(18), 7547-7552. <https://doi.org/10.1073/pnas.1017300108>

Kashiyama, T., Oda, K., Ikeda, Y., Shiose, Y., Hirota, Y., Inaba, K., Makii, C., Kurikawa, R., Miyasaka, A., Koso, T., Fukuda, T., Tanikawa, M., Shoji, K., Sone, K., Arimoto, T., Wada-Hiraike, O., Kawana, K., Nakagawa, S., Matsuda, K., McCormick, F., Aburatani, H., Yano, T., Osuga, Y., & Fujii, T. (2014). Antitumor activity and induction of TP53-dependent apoptosis toward ovarian clear cell adenocarcinoma by the dual PI3K/mTOR inhibitor DS-7423. *PLoS ONE*, *9*(2), e87220. <https://doi.org/10.1371/journal.pone.0087220>

Kunzmann, R., & Hölzel, F. (1987). Karyotype alterations in human ovarian carcinoma cells during long-term cultivation and nude mouse passage. *Cancer Genet Cytogenet*, *28*(2), 201-212. <https://doi.org/10.1016/0165-4608(87)90206-8>

Langdon, S. P., Lawrie, S. S., Hay, F. G., Hawkes, M. M., McDonald, A., Hayward, I. P., Schol, D. J., Hilgers, J., Leonard, R. C., & Smyth, J. F. (1988). Characterization and properties of nine human ovarian adenocarcinoma cell lines. *Cancer Res*, *48*(21), 6166-6172.

Lau, D. H., Lewis, A. D., & Sikic, B. I. (1989). Association of DNA cross-linking with potentiation of the morpholino derivative of doxorubicin by human liver microsomes. *J Natl Cancer Inst*, *81*(13), 1034-1038. <https://doi.org/10.1093/jnci/81.13.1034>

Lounis, H., Mes-Masson, A. M., Dion, F., Bradley, W. E., Seymour, R. J., Provencher, D., & Tonin, P. N. (1998). Mapping of chromosome 3p deletions in human epithelial ovarian tumors. *Oncogene*, *17*(18), 2359-2365. <https://doi.org/10.1038/sj.onc.1202152>

Mitra, A. K., Davis, D. A., Tomar, S., Roy, L., Gurler, H., Xie, J., Lantvit, D. D., Cardenas, H., Fang, F., Liu, Y., Loughran, E., Yang, J., Sharon Stack, M., Emerson, R. E., Cowden Dahl, K. D., M, V. B., Nephew, K. P., Matei, D., & Burdette, J. E. (2015). In vivo tumor growth of high-grade serous ovarian cancer cell lines. *Gynecologic Oncology*, *138*(2), 372-377. <https://doi.org/10.1016/j.ygyno.2015.05.040>

Nozawa, S., Yajima, M., Sasaki, H., Tsukazaki, K., Aoki, D., Sakayori, M., Udagawa, Y., Kobayashi, T., Sato, I., Furusako, S., & et al. (1991). A new CA125-like antigen (CA602) recognized by two monoclonal antibodies against a newly established ovarian clear cell carcinoma cell line (RMG-II). *Jpn J Cancer Res*, *82*(7), 854-861. <https://doi.org/10.1111/j.1349-7006.1991.tb02713.x>

Ohta, H., Sakamoto, H., & Satoh, K. (1998). In vitro effects of gonadotropin-releasing hormone (GnRH) analogue on cancer cell sensitivity to cis-platinum. *Cancer Lett*, *134*(1), 111-118. <https://doi.org/10.1016/s0304-3835(98)00270-5>

Okamura, H., Katabuchi, H., & Ohba, T. (2003). What we have learned from isolated cells from human ovary? *Molecular and Cellular Endocrinology*, *202*(1), 37-45. <https://doi.org/https://doi.org/10.1016/S0303-7207(03)00060-1>

Otte, A., Göhring, G., Steinemann, D., Schlegelberger, B., Groos, S., Länger, F., Kreipe, H. H., Schambach, A., Neumann, T., Hillemanns, P., Park-Simon, T. W., & Hass, R. (2012). A tumor-derived population (SCCOHT-1) as cellular model for a small cell ovarian carcinoma of the hypercalcemic type. *Int J Oncol*, *41*(2), 765-775. <https://doi.org/10.3892/ijo.2012.1468>

Rainey, W. H., Sawetawan, C., Shay, J. W., Michael, M. D., Mathis, J. M., Kutteh, W., Byrd, W., & Carr, B. R. (1994). Transformation of human granulosa cells with the E6 and E7 regions of human papillomavirus. *J Clin Endocrinol Metab*, *78*(3), 705-710. <https://doi.org/10.1210/jcem.78.3.8126145>

Sasano, T., Mabuchi, S., Kuroda, H., Kawano, M., Matsumoto, Y., Takahashi, R., Hisamatsu, T., Sawada, K., Hashimoto, K., Isobe, A., Testa, J. R., & Kimura, T. (2015). Preclinical Efficacy for AKT Targeting in Clear Cell Carcinoma of the Ovary. *Mol Cancer Res*, *13*(4), 795-806. <https://doi.org/10.1158/1541-7786.Mcr-14-0314>

Schilder, R. J., Hall, L., Monks, A., Handel, L. M., Fornace, A. J., Jr., Ozols, R. F., Fojo, A. T., & Hamilton, T. C. (1990). Metallothionein gene expression and resistance to cisplatin in human ovarian cancer. *Int J Cancer*, *45*(3), 416-422. <https://doi.org/10.1002/ijc.2910450306>

Shafren, D. R., Sylvester, D., Johansson, E. S., Campbell, I. G., & Barry, R. D. (2005). Oncolysis of human ovarian cancers by echovirus type 1. *Int J Cancer*, *115*(2), 320-328. <https://doi.org/10.1002/ijc.20866>

Shin, H.-Y., Yang, W., Lee, E.-j., Han, G. H., Cho, H., Chay, D. B., & Kim, J.-h. (2018). Establishment of five immortalized human ovarian surface epithelial cell lines via SV40 T antigen or HPV E6/E7 expression. *PLoS ONE*, *13*(10), e0205297. <https://doi.org/10.1371/journal.pone.0205297>

Simon, W. E., Albrecht, M., Hänsel, M., Dietel, M., & Hölzel, F. (1983). Cell lines derived from human ovarian carcinomas: growth stimulation by gonadotropic and steroid hormones. *J Natl Cancer Inst*, *70*(5), 839-845.

Tajima, K., Hosokawa, K., Yoshida, Y., Dantes, A., Sasson, R., Kotsuji, F., & Amsterdam, A. (2002). Establishment of FSH-responsive cell lines by transfection of pre-ovulatory human granulosa cells with mutated p53 (p53val135) and Ha-ras genes. *Mol Hum Reprod*, *8*(1), 48-57. <https://doi.org/10.1093/molehr/8.1.48>

Uehara, S., Abe, H., Hoshiai, H., Yajima, A., & Suzuki, M. (1984). Establishment and characterization of ovarian endometrioid carcinoma cell line. *Gynecol Oncol*, *17*(3), 314-325. <https://doi.org/10.1016/0090-8258(84)90217-8>

van den Berg-Bakker, C. A. M., Hagemeijer, A., Franken-Postma, E. M., Smit, V. T. H. B. M., Kuppen, P. J. K., Claasen, H. H. V. R., Cornelisse, C. J., & Schrier, P. I. (1993). Establishment and characterization of 7 ovarian carcinoma cell lines and one granulosa tumor cell line: Growth features and cytogenetics. *International Journal of Cancer*, *53*(4), 613-620. <https://doi.org/https://doi.org/10.1002/ijc.2910530415>

Wilson, A. P., Dent, M., Pejovic, T., Hubbold, L., & Radford, H. (1996). Characterisation of seven human ovarian tumour cell lines. *British Journal of Cancer*, *74*(5), 722-727. <https://doi.org/10.1038/bjc.1996.428>

Yamada, K., Tachibana, T., Hashimoto, H., Suzuki, K., Yanagida, S., Endoh, H., Kimura, E., Yasuda, M., Tanaka, T., & Ishikawa, H. (1999). Establishment and characterization of cell lines derived from serous adenocarcinoma (JHOS-2) and clear cell adenocarcinoma (JHOC-5, JHOC-6) of human ovary. *Hum Cell*, *12*(3), 131-138.

Yoshiya, N. (1986). [Establishment of a cell line from human ovarian cancer (undifferentiated carcinoma of FIGO classification) and analysis of its cell-biological characteristics and sensitivity to anticancer drugs]. *Nihon Sanka Fujinka Gakkai Zasshi*, *38*(10), 1747-1753.

Yuan, Y., Kim, W.-H., Han, H. S., Lee, J.-H., Park, H.-S., Chung, J.-K., Kang, S.-B., & Park, J.-G. (1997). Establishment and Characterization of Human Ovarian Carcinoma Cell Lines. *Gynecologic Oncology*, *66*(3), 378-387. <https://doi.org/10.1006/gyno.1997.4785>
